# Incidence, Prevalence, Survival and Mortality of Chronic Lymphocytic Leukaemia/Small Lymphocytic Lymphoma and Waldenström Macroglobulinaemia in Australia

**DOI:** 10.1002/cam4.71582

**Published:** 2026-02-01

**Authors:** Dieu Nguyen, Shalika Bohingamu Mudiyanselage, Dipti Talaulikar, Fei‐Li Zhao, Boxiong Tang, Mostafa Kamal, Lan Gao

**Affiliations:** ^1^ Deakin University Geelong Victoria Australia; ^2^ Australian National University Canberra Australian Capital Territory Australia; ^3^ BeOne Medicines Ltd. Sydney New South Wales Australia; ^4^ BeOne Medicines Ltd. San Carlos California USA

**Keywords:** CLL/SLL, incidence, lymphoid neoplasms, survival, WM

## Abstract

**Background:**

Mature B‐cell neoplasms chronic lymphocytic leukaemia/small lymphocytic lymphoma (CLL/SLL) and waldenström macroglobulinaemia (WM) are highly prevalent in older populations.

**Aims:**

This study quantified the incidence, prevalence and relative survival/mortality rate in Australia for CLL/SS and WM and reported the past trends.

**Materials & Methods:**

All CLL/SLL and WM cases registered from January 2009 to December 2018 in Victoria, Tasmania, Australian Capital Territory and Queensland were identified. Incidence rates over the observed period (2009–2018) were calculated and then projected to 2038 using linear regression. Kaplan–Meier (KM) curves were used to estimate survival rates from 2009 to 2018.

**Results:**

Between 2009 and 2018, the annual age‐standardised incidence rates of CLL/SLL (range, 600.05–887.92 cases per 10^7^ person‐years) and WM (range, 41.48–78.19 cases per 10^7^ person‐years) showed an increasing trend (coefficient: 26.98 [*p* = 0.023] and 3.20 [*p* = 0.009], respectively). A similar trend was seen in age‐standardised prevalence proportions by sex and age group. KM curves showed 10‐year survival rates of 53% (CLL/SLL) and 42% (WM) at the end of the available data period (2018). Differences in survival between sexes were not statistically significant in the log‐rank test, but univariable analysis showed male sex and older age were associated with a higher risk of mortality in both condition.

**Discussion:**

The change in survival over time may reflect disease characteristics and recent advances in treatment.

**Conclusion:**

Given the increasing incidence and relatively high survival of CLL/SLL and WM, strategic planning for the future management is warranted in the context of Australia’s ageing population.

## 1 Introduction

In Western countries, chronic lymphocytic leukaemia/small lymphocytic lymphoma (CLL/SLL; CLL is cancer in the bloodstream; SLL is in the lymph node system [1, 2, 3]) is one of the most common mature B‐cell neoplasms. Lymphoplasmacytic lymphoma (of which 95% of cases are Waldenström macroglobulinaemia [WM]) is a rare type of non‐Hodgkin lymphoma (NHL) characterised by bone marrow infiltration and monoclonal immunoglobulin M gammopathy in the blood that mostly affects adults [1]. Both lymphoid neoplasms are frequently observed in the older population and are considered incurable, albeit with high survival rates. Per current guidelines, patients with asymptomatic CLL/SLL and WM are closely monitored [4, 5, 6, 7]. For patients who require treatment, therapeutic options include anti‐CD20 monoclonal antibody‐based chemoimmunotherapy and newer agents such as Bruton Tyrosine Kinase inhibitors (BTKi) for both CLL and WM and BCL2 inhibitors for CLL [4, 5, 6, 7, 8, 9, 10].

As CLL/SLL and WM are indolent cancers [4, 5], survival rates can be relatively high. International literature suggests a 5‐year overall survival rate of 81% for WM [11] and a 10‐year survival rate of > 60% for patients with CLL ≥ 75 years old in both Germany and the United States (US) [12]. Recent studies using registry data indicated a decreasing trend in the incidence‐based mortality of CLL in Switzerland [2] and WM in women in the US [13]. In Australia, a similar trend was seen in the Australian Cancer Registry report for CLL/SLL and WM, with an increase in the 5‐year survival rate, while the mortality rate was reported as decreasing [14]. For example, the 5‐year observed survival rate of CLL/SLL in Australia increased from 71.4% for the period of 2010–2014 to 74% for the period of 2015–2019 and from 64.4% to 69.5% between the same periods for WM, respectively [15]. However, there were no estimated mortality and survival rates for CLL/SLL and WM over a longer period.

According to the Australian Institute of Health and Welfare (AIHW), NHL is the sixth most diagnosed cancer in Australia [15], with a projected increasing trend in age‐standardised incidence rate for all NHLs (e.g., up to 20.4 cases per 100,000 persons projected in 2021) [15]. To the best of our knowledge, the temporal increasing trend in CLL/SLL and WM incidence rates was reported in an older Australian study using historical lymphoma (lymphoid neoplasms) data from 1982 to 2006 and in the AIHW cancer report using cancer registry data since 2003 [14, 16]. Among all NHLs, CLL/SLL ranked as the most common subtype [16] with an accumulated total age‐standardised incidence rate between 7.6 and 9.6 per 100,000 person‐years for the reported period from 2003 onward. The WM incidence fluctuated between 0.5 and 0.8 per 100,000 persons for the same reporting period [14, 16]. However, the future projection for CLL/SLL and WM incidence was limited in Australia (i.e., 3 years). Similarly, although 16,929 prevalent cases for CLL were recorded by the end of 2018 in the Australian cancer registry [15], no specific rate by sex and age group was reported.

Recent data for incidence, prevalence, survival and mortality of less‐aggressive lymphoid neoplasms with high survival rates, such as CLL/SLL and WM, in Australia are limited. It is essential to investigate the epidemiology of these two subtypes of NHL in the Australian context. Therefore, this study aimed to:

1. Quantify the current incidence rate, prevalence, and mortality indicators of CLL/SLL and WM and
2. Examine the incidence, prevalence, and survival trend of CLL/SLL and WM and predict the trend for the next 30 years, where feasible.

## 2 Methods

### 2.1 Study Design

A population study was performed to examine national administrative data from the four cancer registries (Victoria, Australian Capital Territory, Tasmania and Queensland) of the Australian Cancer Database (ACD) which represent half of the Australian population. At the time of analysis, data were not available from New South Wales, Western Australia, South Australia, and Northern Territory's registries. Descriptive and survival analyses were employed to quantify the current incidence rate, survival, and prevalence of CLL/SLL and WM. Linear regression was used to project and/or predict the incidence and prevalence trend for the observed data and project the 30‐year incidence rate where possible. All analyses were stratified by sex and age group when applicable.

### 2.2 Data Sources and Data Field

In Australia, routine reports of newly diagnosed cancer cases to Australian cancer registries and the ACD, managed by the AIHW, are mandatory. The data were extracted and masked by the AIHW using pre‐defined data fields, including sex, year of diagnosis, age group at diagnosis, *International Statistical Classification of Diseases* (ICD)‐O‐3.2 topography code, ICD‐O‐3.2 histology code, ICD‐10 disease code, rounded survival time (to the nearest multiple of five), age group at death and cause of death (derived from the National Mortality Database).

### 2.3 Study Population

Patients diagnosed with CLL/SLL and WM and registered in the ACD between 01 January 2009 and 31 December 2018 using the *International Statistical Classification of Diseases and Related Health Problems, 10th Revision, Australian Modification* (ICD‐10‐AM) codes were identified. The ICD‐10‐AM codes were C91.1/C83.0 and C88.0 and the ICD‐O‐3.2 histology codes 9823 and 9761 for CLL/SLL and WM, respectively. Due to ACD's data collection methodology, late registration, people with sex recorded as intersex/non‐binary/other, and people whose usual residence at diagnosis was in other territories (Christmas Island, Cocos [Keeling] Islands, Jervis Bay and Norfolk Island) were not included in the dataset. The NSW Cancer Registry granted external ethics approval to conduct this study (ID 2022/ETH01850). In addition, an ethics exemption for this study was approved by the Deakin University Human Ethics Research Committee (DUHERC 2022‐091).

### 2.4 Outcome Measures

Adapting methodologies used in the AIHW reports and previous studies, the incidence rate and prevalence of CLL/SLL and WM were calculated based on the incidence‐based data and the specific Australian mid‐year population size for incidence rate and end‐of‐year population size for prevalence [17]. The incidence rate and prevalence were expressed in a format meeting ethics requirements (i.e., 10,000,000 [or 10^7^] person‐years). Subgroup analysis by age group and sex was undertaken when appropriate. The incidence rate trend for the next 30 years was predicted using the coefficient calculated from least‐squares linear regression [17]. Due to the absence of a cross‐sectional study reporting the point prevalence of CLL/SLL and WM, the 10‐year observed trend of prevalence was examined.

### 2.5 Statistical Analysis

The unit record de‐identified data, aggregated by age group and survival rounded to the nearest multiple of five, were imported into MS Excel, Stata SE17 software (StataCorp., College Station, TX, USA) and Joinpoint Trend Analysis software vs. 5.3.0 [18] for data cleaning and analysis.

Descriptive analyses were performed for the demographic characteristics of the identified cohort. The *χ* ^2^ test was used to compare the differences in characteristics by subgroup. A significance level of 0.05 was set as statistically significant.

Incidence was defined as the number of new patients registered in the ACD during a given year. 10 years prevalence refers to the total number of patients with the disease who are still alive at the end of the follow‐up period (i.e., 2018). Mortality refers to patients who died due to any cause during the study period while survival is the percentage of patients with cancer who are alive for a specified amount of time post‐diagnosis [19]. The details of the annual mortality calculation and the calculation algorithm is presented in Table S1. Given the median survival time of 12.30–13.30 years for patients with CLL/SLL in the literature [20, 21], and the absence of mortality cases among patients diagnosed in previous years (i.e., mortality of patients diagnosed 10–15 years ago in this data set), we did not calculate the annual mortality rate or predict the mortality trend for the observed period. Finally, we calculated the male‐to‐female ratios for incidence rate and prevalence.

We estimated the annual age‐standardised (ASD) incidence rate and age‐standardised prevalence by sex using the direct age standardisation approach that employed the population distribution derived from the ABS provided 2001 Australian standard population [21]. The annual prevalence proportion by specific age group, which represents the disease prevalence within a specific age group of the total population, was calculated by using age‐specific disease cases as the numerator and the person‐years at risk within the age interval as the denominator [22]. Due to the lack of prevalence data in Australia for both CLL/SLL and WM, we employed DisMod II [22], a popular method used in studying disease burden when the dataset is absent or incomplete. This approach allowed for estimation of the proxy prevalence proportions by sex and specific age group for a specific year (i.e., 2009). Age‐standardised prevalence was calculated using the prevalence proportions by sex and specific age group. A 10‐year prevalence was also calculated using the identified 10 years incidence and corresponding mortality cases. A cross‐sectional analysis approach was adopted, capturing a snapshot of incidence and mortality for a particular year. The employed formulae are outlined in the Supporting Method section.

Mid‐year population sizes served as the denominator for calculating incidence rates and prevalence proportions by sex and age group. For the calculation of the 10‐year prevalence, end‐of‐year population data were utilised for the year 2018. Specific population figures by sex and age group were derived to calculate the respective incidence rate and prevalence proportion by sex and age group. The mid‐year and end‐of‐year population sizes were extracted from the Australian Bureau of Statistics [23].

The censoring date was 31 December 2018 in the current dataset. Survival time was defined as the number of days between the diagnosis date and the exit date. Time to death was determined according to the death event documented in the National Death Index. Survival time in days was divided by 365.25 to convert to years.

A least‐squares linear regression was used to capture the time trend of best fit through the 2009–2018 incidence rate and prevalence. *p* < 0.05 was considered statistically significant. This is the approach used by the AIHW for short‐term incidence prediction [17]. Plots of actual incidence rate, age‐standardised incidence rate, age‐standardised prevalence and prevalence proportion for a specific age group over the data duration showed a linear relationship; therefore, the 10‐years age group was included in the model as a categorical variable. The following regression equation was used for the linear prediction of incidence rates for the next 30 years:

$$Y=\beta_{0}+\beta_{1}T$$

with *β*_0_ denoting the intercept, *β*_1_ denoting the coefficients and *T* denoting time (continuous variables) [17]. Assessment of the coefficient of determination (*R* ^2^, *R* squared), a measure to assess the error of the fitted regression model in predicting outcomes, was also estimated. Joinpoint regression limited to a maximum of one joinpoint comprised of two segments model due to the dataset containing only 10 data points [18] was also conducted to provide additional assessment, addressing uncertainty in projecting future incidence trends (i.e., capturing the decline in rate). The result of the joinpoint linear regression is included in the Data S1, along with its final selected model (by using BIC criteria that defaulted in the software [24]) and the associated projections.

Since the dataset contained disease‐related events (e.g., censored status, the death case was identified using time‐to‐event method), Kaplan–Meier (KM) survival curves for overall survival were constructed with a maximum follow‐up of 10 years (since 2009) to estimate overall survival and the associated mortality indicator. A log‐rank test was used to test the difference between subgroups. Relevant commands such as ‘stphplot’ ‘stphtest’ and ‘*estat phtest*’ were used to test the proportionality assumption using Schoenfeld and scaled Schoenfeld residual and the log–log plots during model building and at post estimation [25]. We performed log–log plots for categorical variables (i.e., age group, sex), and Schoenfeld residual plots for all variables. A *p*‐value of < 0.05 was considered statistically significant. Additionally, time‐to‐event analyses were conducted to generate KM curves for each sex, age group and year diagnosed. A Cox proportional hazard model was used to calculate the hazard ratio (HR) for the differences in hazard rate between sex, age group and year of diagnosis. Multivariable analysis stratified by sex and/or age group and interaction terms such as time and the year of diagnosis was employed where appropriate.

## 3 Results

A total of 9002 patients with CLL/SLL and 707 patients with WM were identified in the four cancer registry databases. The populations were predominantly male (CLL/SLL, range 61.04%–64.91%; WM, range 53.85%–70.37%). In total, 2277 patients with CLL/SLL and 192 patients with WM had died by the end of 2018. The most apparent cause of death was not cancer‐related (CLL/SLL, 38.78%; WM, 47.92%), followed by primary cancer (CLL/SLL, 33.64%; WM, 33.33%) and secondary cancer (CLL/SLL, 27.58%; WM, 18.75%) (Table 1).

**TABLE 1:** Summary of case characteristics by count.

	2009	2010	2011	2012	2013	2014	2015	2016	2017	2018	*p*
CLL/SLL, *n*	696	783	761	749	761	811	961	1119	1253	1108	—
WM, *n*	64	52	57	65	54	67	66	77	90	115	
Sex											
CLL/SLL (*n* = 9002)											
Male, *n*	438	482	477	481	494	516	614	683	770	703	0.739
%	62.93	61.56	62.68	64.22	64.91	63.63	63.89	61.04	61.45	63.45	
Female, *n*	258	301	284	268	267	295	347	436	483	405	
%	37.07	38.44	37.32	35.78	35.09	36.37	36.11	38.96	38.55	36.55	
WM (*n* = 707)											
Male, *n*	38	35	35	35	38	42	39	42	61	71	0.581
%	59.38	67.31	61.4	53.85	70.37	62.69	59.09	54.55	67.78	61.74	
Female, *n*	26	17	22	30	16	25	27	35	29	44	
%	40.63	32.69	38.6	46.15	29.63	37.31	40.91	45.45	32.22	38.26	
Age group											
CLL/SLL (*n* = 9002)											
< 50 years, *n* ^a^	49	47	35	47	41	49	52	52	54	50	0.224
%	7.04	6	4.6	6.28	5.39	6.04	5.41	4.65	4.31	4.51	
50–59 years, *n*	107	123	116	104	119	116	123	147	184	139	
%	15.37	15.71	15.24	13.89	15.64	14.3	12.8	13.14	14.68	12.55	
60–69 years, *n*	182	224	209	214	235	238	277	314	336	302	
%	26.15	28.61	27.46	28.57	30.88	29.35	28.82	28.06	26.82	27.26	
70–79 years, *n*	196	205	223	206	194	248	284	344	389	356	
%	28.16	26.18	29.3	27.5	25.49	30.58	29.55	30.74	31.05	32.13	
80–89 years, *n*	139	150	144	151	144	131	196	221	229	219	
%	19.97	19.16	18.92	20.16	18.92	16.15	20.4	19.75	18.28	19.77	
90+ years, *n*	23	34	34	27	28	29	29	41	61	42	
%	3.3	4.34	4.47	3.6	3.68	3.58	3.02	3.66	4.87	3.79	
WM (*n* = 707)											
< 60 years, *n* #	6	9	7	11	8	10	10	12	10	11	0.711
%	9.38	17.31	12.28	16.92	14.81	14.93	15.15	15.58	11.11	9.57	
60–69 years, *n*	19	10	15	15	17	20	15	17	18	31	
%	29.69	19.23	26.32	23.08	31.48	29.85	22.73	22.08	20	26.96	
70–79 years, *n*	21	17	17	16	13	21	24	22	29	49	
%	32.81	32.69	29.82	24.62	24.07	31.34	36.36	28.57	32.22	42.61	
80+ years, *n*	18	16	18	23	16	16	17	26	33	24	
%	28.13	30.77	31.58	35.38	29.63	23.88	25.76	33.77	36.67	20.87	
Cause of death^a^											
CLL/SLL (*n* = 2277)											
CLL/SLL, *n*	136	129	111	82	82	66	59	42	38	21	0.019
%	38.86	37.94	36.27	28.98	34.45	30.84	32.07	26.58	25.33	38.89	
Secondary cancer, *n*	90	79	79	102	58	63	51	50	41	15	
%	25.71	23.24	25.82	36.04	24.37	29.44	27.72	31.65	27.33	27.78	
Not cancer related, *n*	124	132	116	99	98	85	74	66	71	18	
%	35.43	38.82	37.91	34.98	41.18	39.72	40.22	41.77	47.33	33.33	
WM (*n* = 192)											
WM, *n*	15	9	5	9	5	9	6	—^b^	—^b^	—^b^	0.055
%	37.5	34.62	21.74	37.5	27.78	56.25	46.15	NR	NR	NR	
Secondary cancer, *n*	7	—^b^	6	—^b^	—^b^	—^b^	—^b^	—^b^	—^b^	—^b^	
%	17.5	NR	26.09	NR	NR	NR	NR	NR	NR	NR	
Not cancer related, *n*	18	14	12	11	12	—^b^	6	6	9	—^b^	
%	45	53.85	52.17	45.83	66.67	NR	46.15	46.15	64.29	NR	

*Note:* Percentages may not total 100% due to rounding.

Abbreviations: CLL/SLL, chronic lymphocytic leukaemia/small lymphocytic lymphoma; NR, not reported; WM, Waldenström macroglobulinaemia.

^a^ The death cases presented in this table are the death case by the year of diagnosis. For example, the *n* = 21 case of the year 2018 in CLL/SLL patients represented the death case of patients who diagnosed in the year 2018 only.

^b^ Insufficient cases (*n* < 5) were detected for the analysis.

### 3.1 Incidence Rate and 30‐Year Incidence Trend

#### 3.1.1 CLL/SLL

**FIGURE 1:**
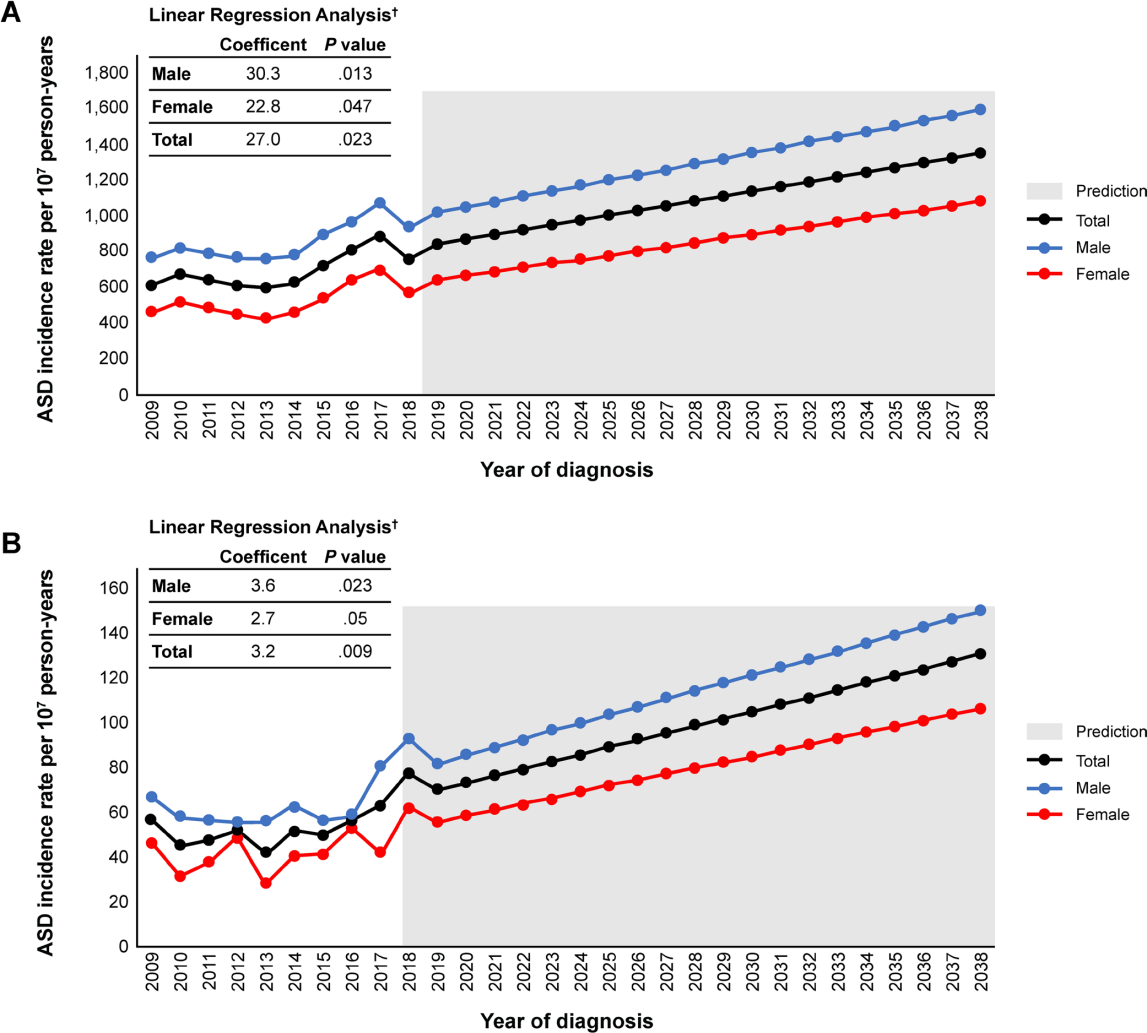
Linear prediction of annual (A) CLL/SLL and (B) WM age‐standardised incidence rates over 30 years (2009–2038). CLL/SLL, chronic lymphocytic leukaemia/small lymphocytic lymphoma; WM, Waldenström macroglobulinaemia. ^†^Coefficients determined by a least‐squares linear regression analysis of incidence data from 2009 to 2018. *p* < 0.05 was considered statistically significant.

The annual CLL/SLL incidence rate ranged from 659.10–1029.86 (crude) and 600.05–887.92 (ASD) cases per 10^7^ person‐years in 2009–2017. The rate slightly decreased in 2018 to 894.20 (crude) and 761.01 (ASD) per 10^7^ person‐years (Table 2). Incidence rates by sex had a similar temporal trend, but males had a slightly higher annual incidence rate than females (incidence rate ratio, males vs. females, 1.60–1.88). The incidence rate was lowest in the under 40–49 age group and higher in the ≥ 80 age group (Table S2). Linear regression analysis suggested that the total CLL/SLL ASD incidence rate is predicted to increase over the next 30 years (Figure 1A). A coefficient of 30.29 (*p* = 0.013), 22.75 (*p* = 0.047) and 26.98 (*p* = 0.023) was detected for males, females, and all patients with CLL/SLL, respectively, using the 10‐year data (Table S3). Joinpoint regression analysis using one segment model showed an average annual percentage change (AAPC) of 3.56%, 4.39% and 3.91% for males, females and total CLL/SLL patients. The magnitude of the change was comparable between linear regression and joinpoint regression results. Although a non‐statistically significant decreased trend between 2009 and 2014 was seen in the joinpoint model with two segments, this model was not selected due to its higher weighted BIC (Table S3; Figure S1A). Using linear regression results, the annual ASD incidence rate was predicted to reach 1351.90 cases per 10^7^ person‐years in 2038 (Table S4; Figure 1A).

**TABLE 2:** Incidence rates and incidence trend by sex over the observed period (2009–2018).

Incidence rate, cases per 10^7^ person‐years											Incidence trend		
Year	2009	2010	2011	2012	2013	2014	2015	2016	2017	2018	Intercept *β*_0_ (SD)	Coefficient *β*_1_ (SD)	*R* ^2^
CLL/SLL													
Male	833.86	903.07	881.41	872.09	879.18	903.62	1058.56	1156.31	1278.47	1146.18	744.71 (54.82)*	44.83 (8.84)*	0.763
Female	486.13	557.57	518.17	479.4	468.5	508.37	587.53	723.45	786.15	647.21	428.24 (53.23)*	26.91 (8.84)*	0.552
Total	659.1	729.34	698.64	674.42	672.38	704.41	820.92	937.7	1029.86	894.2	585.89 (53.58)*	35.67 (8.64)*	0.681
ASD male	767.52	823.51	791.90	769.89	763.39	784.13	895.14	968.55	1066.02	939.94	685.22 (60.11)*	30.29 (9.20)*	0.607
ASD female	467.25	520.45	484.57	442.42	427.94	463.22	536.07	648.33	698.47	571.31	396.02 (61.90)*	22.75 (9.48)*	0.452
ASD total	620.76	674.42	640.34	610.74	600.05	628.45	720.47	815.45	887.92	761.01	542.42 (60.66)*	26.98 (9.29)*	0.547
WM													
Male	72.34	65.57	64.66	63.44	67.61	73.52	67.21	71.07	101.22	115.67	53.27 (8.90)*	4.17 (1.43)*	0.514
Female	48.99	31.49	40.14	53.66	28.07	43.07	45.7	58.06	47.18	70.28	33.92 (7.37)^a^	2.32 (1.19)*	0.322
Total	60.61	48.43	52.32	58.52	47.7	58.18	56.36	64.5	73.94	92.75	43.57 (6.71)*	3.23 (1.08)*	0.527
ASD male	66.54	57.98	56.77	55.12	55.98	62.16	56.54	57.80	80.37	92.61	42.40 (8.08)*	3.59 (1.24)*	0.545
ASD female	46.01	31.82	37.01	49.17	26.92	40.32	41.19	52.74	41.75	62.80	26.68 (7.35)*	2.66 (1.12)	0.444
ASD total	56.53	45.46	47.62	52.39	41.80	51.80	49.87	55.89	62.99	78.19	34.79 (5.89)*	3.20 (0.90)*	0.643

Abbreviations: ASD, age‐standardised using the 2001 Australian standard population; CLL/SLL, chronic lymphocytic leukaemia/small lymphocytic lymphoma; SD, standard deviation; WM, Waldenström macroglobulinaemia.

* *p* < 0.05.

^a^ *p* = 0.087.

#### 3.1.2 WM

For WM, results showed a stable overall incidence rate between 2009 and 2015, followed by an increasing trend between 2016 and 2018 (Table 2; Figure 1B). While the incidence rate in females fluctuated between 2009 and 2015, the rate increased in 2016 and was highest in 2018 (ASD: 62.80, Crude: 70.28 cases per 10^7^ person‐years). Meanwhile, the rate in males was stable between 2009 and 2016 and increased sharply between 2017 and 2018. The incidence rate ratio of WM (males vs. females) ranged from 1.22 to 2.41 for the observed period. The linear regression model predicted a statistically significant ascending trend for the WM ASD‐incidence rate during the 10‐year period (males: coefficient 3.59, *p* = 0.023; females: 2.66, *p* = 0.050; total: 3.20, *p* = 0.009) (Table 2; Figure 1B). Joinpoint regression estimated the AAPC of 4.52%, 3.96% and 4.39% for males, females and total WM with non‐statistically significant evidence to support the decrement between 2009 and 2015 (Table S3; Figure S1B). Similar to CLL/SLL, the past trend was comparable between linear regression results and joinpoint regression results; thus, the ASD incidence rate of WM will reach 130.83 cases per 10^7^ person‐years in 2038 (Table S4; Figure 1B).

### 3.2 Prevalence Rates and the Observed Trend

#### 3.2.1 CLL/SLL

For the observed period of 2009–2018, the CLL/SLL 10 years prevalence was 5380.08 per 10^7^ persons at the end of 2018. The prevalence was higher in males than in females, with a prevalence ratio of 1.70. During the 10 year observed period, the increasing trend in the prevalence for males vs. females was also determined by linear regression analysis (males, prevalence proportion: coefficient 88.02, *p* < 0.001 and males, ASD prevalence: 41.92, *p* = 0.010; females, prevalence proportion: 44.27, *p* < 0.001 and females, ASD prevalence: 27.50, *p* = 0.053) (Tables S5 and S6).

#### 3.2.2 WM

A point prevalence of 412.07 per 10^7^ persons was estimated for patients with WM at the end of the observed period (2009–2018). Similar to CLL/SLL, the prevalence was higher in males than in females (ratio, 1.51).

Linear regression suggested a coefficient of 10.29 (*p* = 0.016) and 4.85 (*p* = 0.020) for males by age group specific and by ASD prevalence respectively and a coefficient of 4.03 (*p* = 0.106) and 3.52 (*p* = 0.055) for females by age group specific and by ASD prevalence respectively, suggesting an increasing trend in the age‐standardised prevalence proportion by sex and age group over 10 years (Tables S5 and S6).

### 3.3 Survival Estimates and Mortality Indicator

Due to insufficient follow‐up in the data (median overall survival time was not estimable in the survival analysis) and the median survival time of CLL/SLL being 13.15–13.30 years in the literature [20, 26], the annual incidence‐based mortality rate was not calculated.

#### 3.3.1 CLL/SLL

**FIGURE 2:**
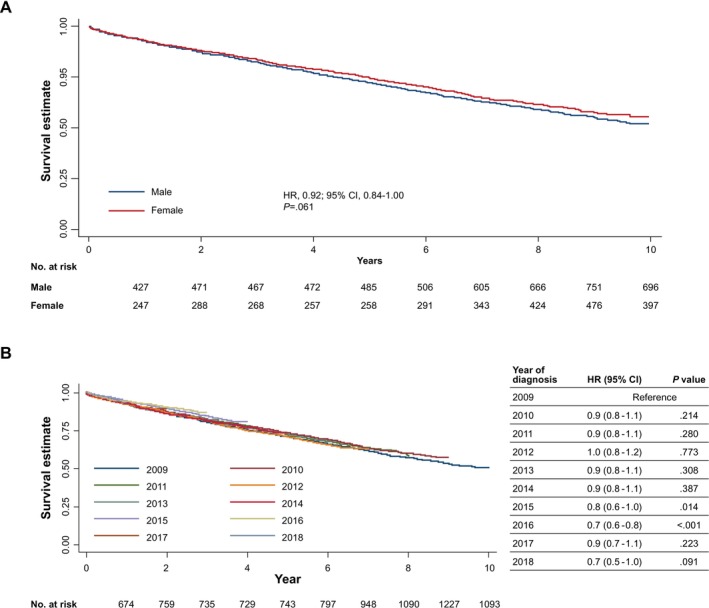
Kaplan–Meier survival estimate by (A) sex and (B) year of diagnosis for CLL/SLL with 10 years of follow‐up. Curves were generated by Kaplan–Meier survival analyses (HR 0.92, 95% CI 0.84–1.00) and evaluated by the log‐rank test (*p* = 0.061 significance at *p* < 0.05). CI, confidence interval; CLL/SLL, chronic lymphocytic leukaemia/small lymphocytic lymphoma; HR, hazard ratio.

The KM analysis estimated that approximately 53% of patients with CLL/SLL were still alive at the end of the 10‐year analysis period (2009–2018), indicating a cumulative mortality rate of 47%. Females had a slightly better survival rate (Figure 2A); however, the log‐rank test for the differences between sexes was not statistically significant (*p* = 0.061). There was a statistically significant difference in survival based on years of diagnosis and age groups for patients (*p* = 0.038 and *p* < 0.001, respectively) (Figure 2B; Figure S2). Results from Cox proportional regression analyses indicated a significantly higher mortality rate in older patients. For example, a HR of 2.00 (95% CI, 1.63–2.46; *p* < 0.001) was detected in those aged 60–69 versus < 60 years.

#### 3.3.2 WM

**FIGURE 3:**
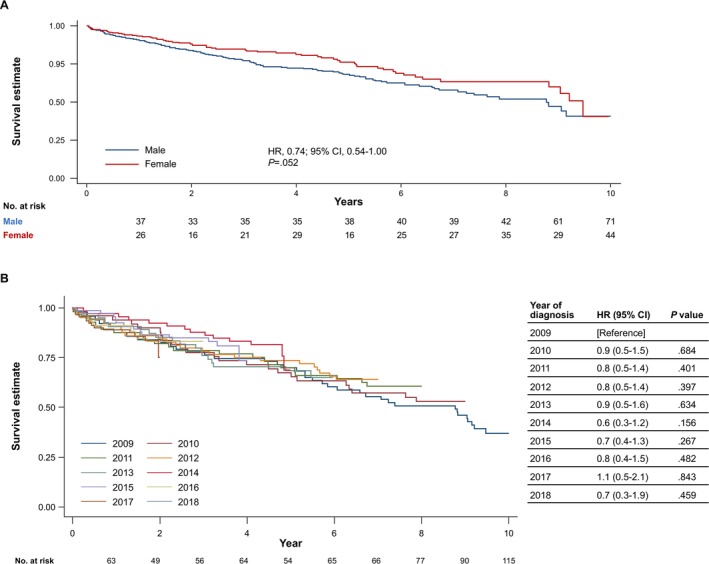
Kaplan–Meier survival estimate by (A) sex and (B) year of diagnosis for WM with 10 years of follow‐up. Curves were generated by Kaplan–Meier survival analyses (HR 0.74, 95% CI 0.54–1.00) and evaluated by the log‐rank test (*p* = 0.052, significance at *p* < 0.05). CI, confidence interval; HR, hazard ratio; WM, Waldenström macroglobulinaemia.

In patients with WM, the KM survival analysis estimated that 42% of patients were alive at the end of the 10‐year follow‐up period. The log‐rank test for the difference in survival rates between sexes and year of diagnosis was not statistically significant (*p* = 0.052 and *p* = 0.917, respectively) (Figure 3; Table 3). However, differences in survival rates between age groups were evident (*p* < 0.001; Figure S2). Cox proportional regression univariable analyses for overall survival suggested a statistically significant difference in mortality rate between patients aged < 70 years versus aged 70–79 years (HR, 1.63; 95% CI, 1.06–2.52, *p* = 0.028) and ≥ 80 (HR, 5.76; 95% CI, 3.94–8.41; *p* < 0.001) (Table 3).

**TABLE 3:** Hazard ratio of CLL/SLL and WM by sex, age group, and year of diagnosis.

		*n* (%)	Model 1‐ univariate analysis				Model 2‐ multivariable analysis of all‐cause mortality				Model 3‐ multivariable analysis of cancer specific death			
			HR	95% CI		*p*	HR	95% CI		*p*	HR	95% CI		*p*
CLL/SLL														
Age group	< 60	1754 (19.48)	Reference				Reference (stratified by age group)				Reference (stratified by age group)			
	60–69	2531 (28.12)	2.00	1.63	2.46	< 0.001								
	70–79	2645 (29.38)	4.51	3.72	5.47	< 0.001								
	80–89	1724 (19.15)	12.17	10.06	14.72	< 0.001								
	90+	348 (3.87)	27.06	21.53	34.02	< 0.001								
Sex	Male	5658 (62.85)	Reference				Reference				Reference			
	Female	3344 (37.15)	0.92	0.84	1.00	0.061	0.73	0.67	0.80	< 0.001	0.65	0.58	0.74	< 0.001
Year	2009–18	9002 (100)	0.98	0.92	1.04	0.441	0.97	0.95	0.99	< 0.001	0.90	0.88	0.92	< 0.001
Year (in tvc model)							0.97	0.95	0.98	< 0.001	0.90	0.88	0.92	< 0.001
Time × year of diagnosis interaction							1.00	0.99	1.01	0.748	0.99	0.98	1.01	0.336
Pre‐model proportional hazard assumption test										0.897				0.642
Post model estimation										0.979				0.643
WM														
Age group	< 70	271 (38.33)	Reference				Reference (stratified by age group)				Reference (stratified by age group)			
	70–79	229 (32.39)	1.63	1.06	2.52	0.028								
	80+	207 (29.28)	5.76	3.94	8.41	< 0.001								
Sex	Male	71 (61.74)	Reference				Reference							
	Female	44 (38.26)	0.74	0.54	1.00	0.053	0.69	0.50	0.93	0.017	0.74	0.49	1.12	0.154
Year	2009–2018	699 (100)	0.97	0.95	0.99	0.003	0.97	0.91	1.03	0.292	0.91	0.84	0.99	0.024
Year (in tvc model)							0.96	0.90	1.03	0.259	0.91	0.84	0.99	0.024
Time × year of diagnosis interaction							0.99	0.95	1.03	0.636	0.98	0.93	1.03	0.406
Pre‐model proportional hazard assumption test*										0.724				0.541
Post model estimation*										0.745				0.553

Abbreviations: CI, confidence interval; CLL/SLL, chronic lymphocytic leukaemia/small lymphocytic lymphoma; HR, hazard ratio; tvc, Inbuilt option in STATA to capture time interaction term with a chosen variable; WM, Waldenström macroglobulinaemia.

* *p*‐value > 0.05 indicates non‐violation of the proportional hazard assumption and the model is a good fit.

## 4 Discussion

This study quantified the incidence, prevalence, and estimated survival and mortality indicator of CLL/SLL and WM using data from the four cancer registries in Australia (Victoria, Australian Capital Territory, Queensland and Tasmania). Results suggested that from 2009 to 2018, the incidence rate and prevalence for CLL/SLL and WM generally increased. The high survival rate observed during the 10‐year period may reflect the impact of anti‐CD20 monoclonal antibody therapy (single agent or in combination) and the new BTKi on the management of these two lymphoid neoplasms in the Australian population [4, 5]. Given the population covered by these four cancer registries represented half of the Australian population, calculated measures are likely to be generalisable to the wider population.

Patients with CLL/SLL or WM tend to be older and male [27, 28, 29]. Evidence from this study and international studies suggests that incidence is dominant in the ≥ 50 age group for CLL and ≥ 60 for WM [27, 28, 29]. The rising incidence trend detected in this study was compatible with international evidence. For example, evidence from the recent global burden of disease study for CLL suggested an increased trend of CLL in the Australasia region from 2.99 per 100,000 people in 1990 to 5.2 per 100,000 people in 2019 with an annual percentage change of 1.55% [2, 27]. An increased trend was also reported for WM in a small cohort in Minnesota in the US (from 0.24 per 100,000 person‐years to 0.56 per 100,000 person‐years) and in the Health Insurance Review and Assessment Service database in Korea (from 0.03 per 100,000 person‐years in 2003 to 0.10 per 100,000 person‐years in 2016) [29, 30]. Although there is no explanation for the change in CLL/SLL and WM incidence rates over time, the increased incidence trend detected in both conditions at the end of the observed period (i.e., after 2016) might be attributed to changes in the approach for diagnosis and management of CLL/SLL [4] and the publication of the first Australian WM clinical practice guideline in early 2017 [31]. Notably, the obtained *R* ^2^‐values for the coefficient trend detected during the observed period suggested a linear relationship, and the regression model for incidence rate had a reasonable fit (e.g., *R* ^2^ line between 0.5 and 1 for male and total persons), suggesting good interaction between year of diagnosis and incidence rate of CLL/SLL and WM.

Regarding the cause of death attributable to comorbidities in the older patient cohort, this cohort had a high proportion of non‐cancer‐related deaths for both conditions (CLL/SLL, 33.33%–47.33%; WM, 45%–66.67%). The CLL rate was lower than the rate reported in the literature identified from the 20‐year follow‐up data in the Mayo Clinic CLL Database (48.9%) [32]. Similarly, the rate of non‐cancer‐related death for WM was quite similar to the rate reported in international literature (approximately 50% in the US population) [33]. Due to the absence of comorbidity information in the dataset, the rate of death attributable to specific comorbidities could not be quantified. Previous literature shows that comorbidities such as cardiovascular diseases (i.e., atrial fibrillation, heart failure) [34], dementia, and haemorrhage‐related conditions (due to the use of anticoagulants and antiplatelets) [35] are highly prevalent in patients with CLL or WM [36]. For example, the incidence of major haemorrhage in patients with CLL was reported to be 1.9 per 100 person‐years, with a 5‐year cumulative incidence rate of 7.3% [35]. In addition, patients with CLL who have comorbidities (according to the Charlson Comorbidity Index) have a 37%–65% higher mortality risk compared to patients with CLL with no comorbidity (HR, 1.35; 95% CI, 1.25–1.45 vs. HR, 1.65; 95% CI, 1.56–1.75 for patients with > 2 comorbidities) [36]. The high prevalence of comorbidities in patients with CLL and WM may partially explain the increased probability of non‐cancer‐related mortality in these patients.

In 2019–2020, cancer and other neoplasms were the third highest healthcare expenditure in Australia (AUS$12.1 billion) [37]. The direct lifetime financial costs of individuals diagnosed with blood cancer in 2019 were estimated at AUS$3.4 billion [38]. This study and the international literature suggest that patients with CLL/SLL or WM have medium to high survival rates [11, 12, 36], indicating a relatively long duration of survivorship. As CLL/SLL and WM are incurable diseases that require long‐term follow up with a high probability of remission, relapse and/or secondary malignancies and treatment‐related complications (i.e., neutropenia after zanubrutinib treatment) [4, 5], it is not surprising that among cancers with a high 5‐year survival rate, NHL had the highest mean annual health service costs per individual (AUS$24397). Similarly, among cancers considered to have medium survival rates, leukaemia (comprised of CLL and other leukaemia) had the second highest mean annual costs (AUS$29158/individual) [39].

There are several strengths of the study. First, it was one of few in Australia and internationally that used population‐level administrative data to examine the trend of incidence rate and prevalence proportion and to estimate the survival and mortality indicator of CLL/SLL and WM over time. The study included a modelling component that used 10‐year data of incidence and mortality to determine the trends in the past. The identified coefficient for this trend was then used to predict the future 30‐year trend for incidence and to identify the direction of the prevalence trend. Using past trends to predict future trends is deemed one of the best approaches for projecting what will happen if nothing changes in practice [40]. This approach was done previously with cross‐sectional data to predict the future 25‐year mean body mass index and the prevalence of obesity in Australia [40]. Although the analysis could not project the trend for future prevalence due to limitations of mortality data and the absence of CLL/SLL and WM point prevalence in Australia, the predicted coefficient of prevalence proportional by sex within specific age groups and the age‐standardised prevalence might act as proxy evidence to suggest an increase in the annual prevalence over time. Finally, this study includes a large sample size, covering approximately half of the Australian population. This can provide a reliable measure for incidence and survival at the country level and provides an accurate estimation for the incidence trend over the next 30 years as well as the observed survival and associated hazard ratios.

### 4.1 Limitations

Despite the use of a validated approach, this study has several limitations. Firstly, it cannot be ensured that all cases of WM and CLL/SLL and the loss to follow up were captured, as the analysis relied on registry data that contains all new cancer diagnoses in a single year based on ICD‐10 codes. An earlier Spanish study acknowledged a similar issue when comparing the CLL/SLL incidence using different datasets (i.e., registry datasets using ICD‐10 vs. other means of diagnosis, such as pathology). The lack of this detailed clinical information might restrict performing further analyses. Future studies using other datasets, such as linked datasets containing additional information on disease progression and treatment status, are recommended. These studies could provide valuable insights into the incidence by subgroup, thereby informing policy and practice, especially in workforce and health care planning. In addition, selection bias and undetected confounding variables are unavoidable in this type of research, as exemplified by the study which only included patients with CLL/SLL or WM identified by the relevant ICD‐10 code without additional clinical information (e.g., clinical notes and/or pathology data) [41]. However, the greatest strength of using registry data is disease surveillance in the real world [41]. Furthermore, in calculating the mortality case to inform the prevalence calculation for this study, systematic measurement bias could affect findings as the analysis assumed that everyone was diagnosed at the same time. Lastly, it is important to recognise the limitation of the least‐squares linear regression in estimating the past trend and predicting the future, in which the model was unable to capture the change within cohort demographic characteristics (i.e., age) and the change that was associated with new intervention or change in practice. Therefore, caution must be used when applying generated coefficients to project long‐term future incidence and prevalence. The future rate might only be accurate in the short term. However, it was reassuring that a clear trend was shown for all rates in both conditions with high *R* ^2^ (i.e., > 0.80 for the prevalence proportion by age group model), suggesting that the model was a good fit. Furthermore, the identified trend was similar to the trend over time observed internationally [29] and the past trend reported by the AIHW (the government agency for collecting and analysing disease/health data) [15].

## 5 Conclusion

Incidence rate and prevalence of CLL/SLL and WM have been increasing over the last decade. The linear regression model predicted an increase in CLL/SLL and WM incidence and prevalence over the observed period. A lower mortality risk was observed in younger age groups and females, while a high survival rate was observed over a 10‐year period for both diseases, probably due to the nature of the cancers and recent advances in treatment. With the increasing incidence trend over time and high survival rates, strategic policies are required to address these two indolent conditions, especially regarding the healthcare system's readiness to serve the ageing population.

## Author Contributions

**Dieu Nguyen:** conceptualization (equal), data curation (equal), formal analysis (equal), methodology (equal), writing – original draft (equal), writing – review and editing (equal). **Shalika Bohingamu Mudiyanselage:** formal analysis (equal), writing – original draft (equal), writing – review and editing (equal). **Dipti Talaulikar:** writing – original draft (equal), writing – review and editing (equal). **Fei‐Li Zhao:** writing – original draft (equal), writing – review and editing (equal). **Boxiong Tang:** writing – original draft (equal), writing – review and editing (equal). **Mostafa Kamal:** data curation (equal), formal analysis (equal), writing – original draft (equal), writing – review and editing (equal). **Lan Gao:** conceptualization (equal), data curation (equal), formal analysis (equal), methodology (equal), project administration (lead), writing – original draft (equal), writing – review and editing (equal).

## Funding

This work was funded by the Institute for Health Transformation Category 1 Seed Funding Grant, Deakin University and a research grant from BeOne Medicines Ltd.

## Ethics Statement

An ethics exemption for this study was approved by the Deakin University Human Ethics Research Committee (DUHERC 2022‐091).

## Conflicts of Interest

L.G. and D.N. received a seed funding grant from the Institute for Health Information and Deakin University and a research grant from BeOne Medicines Ltd. BeOne Medicines Ltd. did not hold any influence over the publication of results. The results were reported with ethics approval and adherence to the Australian Code for the Responsible Conduct of Research in an appropriate format. All the authors and the data custodian have given consent for publication as stated in the ethics application and ethics approval.

## Supporting information


**Data S1:** cam471582‐sup‐0001‐DataS1.docx.

## Data Availability

The data that support the findings of this study are available upon request from AIHW. However, restrictions apply to the availability of these data in conjunction with the Privacy Act and the Australian Code for the Responsible Conduct of Research in appropriate format.
