# Supplementary material for: Incidence, Prevalence, Survival and Mortality of Chronic Lymphocytic Leukaemia/Small Lymphocytic Lymphoma and Waldenström Macroglobulinaemia in Australia
Source: Cancer Med. 2026 Feb 1;15(2):e71582. doi: 10.1002/cam4.71582 (PMC12861573; doi:10.1002/cam4.71582)
Supplement: Supplementary file 1 — Data S1: cam471582‐sup‐0001‐DataS1.docx. [file CAM4-15-e71582-s001.docx]

**Supporting Information: Incidence, prevalence, survival & mortality of chronic lymphocytic leukemia/small lymphocytic lymphoma & Waldenström macroglobulinemia in Australia**

**Authors:** Dieu Nguyen,^1^ Shalika Bohingamu Mudiyanselage,^1^ Dipti Talaulikar,^2^ Fei-Li Zhao,^3^ Boxiong Tang,^4^ Mostafa Kamal,^1^ Lan Gao^1^

**Affiliations:** ^1^Deakin University, Geelong, VIC, Australia; ^2^Australian National University, Canberra, ACT, Australia; ^3^BeOne Medicines Ltd., Sydney, NSW, Australia; ^4^BeOne Medicines Ltd., San Carlos, CA, USA

**Table of Contents**

[Supporting method 2](#_Toc189142502)

[Supporting Table 1. Matrix of death by year of DM and assumed year of death 4](#_Toc189142503)

[Supporting Table 2. Incidence rate per 10^7^ person-years by age group 6](#_Toc189142504)

[Supporting Table 3. Table of coefficient results for incidence rate examining the trend over 10 years observed data 9](#_Toc189142505)

[Supporting Table 4. Thirty-year incidence prediction for CLL/SLL and WM 11](#_Toc189142506)

[Supporting Table 5. Prevalence proportion per 10^7^ persons-years by sex and age group for CLL/SLL and WM between 2009-2018 13](#_Toc189142507)

[Supporting Table 6. Linear regression by age group for prevalence proportion of CLL/SLL and WM (per 10^7^ person-years) examining the trend over 10 years observed data 15](#_Toc189142508)

[Supporting Figure 1A. Joinpoint regression result CLL/SLL 18](#_Toc189142509)

[Supporting Figure 1B. Joinpoint regression result WM 19](#_Toc189142510)

[Supporting Figure 2. Kaplan-Meier survival estimate by age group for CLL/SLL with 10 years of follow-up 20](#_Toc189142511)

[Supporting Figure 3. Kaplan-Meier survival estimate by age group for WM with 10 years of follow-up 21](#_Toc189142512)

[Supporting Figure 4A. Schoenfeld and scaled Schoenfeld residual test for the proportionality assumption (CLL/SLL) 22](#_Toc189142513)

[Supporting Figure 4B. Schoenfeld and scaled Schoenfeld residual test for the proportionality assumption (WM) 23](#_Toc189142514)

# Supporting method

The below formulae were used for incidence, prevalence and prevalence proportion calculations:

Incidence rate$IR=\frac{number of people who developed the disease in 1 year}{average (mid-year) population}$(1)

Point prevalence $PP=\frac{total number of diseased people still alive in a given year}{total number of people in the population}$ (1)

In this paper, the 2018 point prevalence refers to the 10-year prevalence at the end of 2018 where the numerator is the total cumulative incidence cases minus total cumulative mortality cases recorded at the end of 2018. The denominator is the Australian end-of-year (2018) population reported by ABS (2)

Prevalence ratio $PR=\frac{prevalence rate (A)}{prevalence rate (B)}$ (1)

Incidence rate ratio $IRR=\frac{incidence rate (A)}{incidence rate (B)}$ (1)

Prevalence proportion = 0.5[(C_a_+C_a+1_)/PY_a_] (3)

In this formula, PY_a_ = (S_a_ + S_a+1_+C_a_+C_a+1_) is the person-year that refers to the general people in the population for the specific age group, S_a_ is the number of people unaffected by the disease at age a, C_a_ is the number of diseased people at age a. The formula was used under the assumption that the prevalence of CLL/SLL and WM is zero at age zero while the distribution of prevalence is binominal (3).

Direct standardisation ASR=∑Niri/∑Ni

In this formula, ri is the event rate of each age group of the study population, Ni is the standard population size for each age group

# Supporting Table 1. Matrix of death by year of DM and assumed year of death

| **Year of death*** | **Number of deaths by year** | | | | | | | | | | **Total no of death per year** |
| --- | --- | --- | --- | --- | --- | --- | --- | --- | --- | --- | --- |
|  | **2009** | **2010** | **2011** | **2012** | **2013** | **2014** | **2015** | **2016** | **2017** | **2018** |  |
| Mid 2009 | New DM | NA | NA | NA | NA | NA | NA | NA | NA | NA | - |
| 2009 | X | NA | NA | NA | NA | NA | NA | NA | NA | NA | X |
| Mid 2010 | Y | New DM | NA | NA | NA | NA | NA | NA | NA | NA | - |
| 2010 | X | X | NA | NA | NA | NA | NA | NA | NA | NA | 2X+Y |
| Mid 2011 | Y | Y | New DM | NA | NA | NA | NA | NA | NA | NA | - |
| 2011 | X | X | X | NA | NA | NA | NA | NA | NA | NA | 3X+2Y |
| Mid 2012 | Y | Y | Y | New DM | NA | NA | NA | NA | NA | NA | - |
| 2012 | X | X | X | X | NA | NA | NA | NA | NA | NA | 4X+3Y |
| Mid 2013 | Y | Y | Y | Y | New DM | NA | NA | NA | NA | NA | - |
| 2013 | X | X | X | X | X | NA | NA | NA | NA | NA | 5X+4Y |
| Mid 2014 | Y | Y | Y | Y | Y | New DM | NA | NA | NA | NA | - |
| 2014 | X | X | X | X | X | X | NA | NA | NA | NA | 6X+5Y |
| Mid 2015 | Y | Y | Y | Y | Y | Y | New DM | NA | NA | NA | - |
| 2015 | X | X | X | X | X | X | X | NA | NA | NA | 7X+6Y |
| Mid 2016 | Y | Y | Y | Y | Y | Y | Y | New DM | NA | NA | - |
| 2016 | X | X | X | X | X | X | X | X | NA | NA | 8X+7Y |
| Mid 2017 | Y | Y | Y | Y | Y | Y | Y | Y | New DM | NA | - |
| 2017 | X | X | X | X | X | X | X | X | X | NA | 9X+8Y |
| Mid 2018 | Y | Y | Y | Y | Y | Y | Y | Y | Y | New DM | - |
| 2018 | X | X | X | X | X | X | X | X | X | X | 10X+9Y |
| Mid 2019 | Y | Y | Y | Y | Y | Y | Y | Y | Y | Y | - |

^†^ Assumes that all patients are diagnosed at mid-year; X is mortality cases in the first half year, and Y is mortality of the second half year within a yearly interval; DM: New diagnosed and being managed case

Technical note: Annual mortality cases are defined as deaths occurring in a specific year (i.e., 2018)(4). Annual cancer mortality cases were identified using the partitioning cancer mortality approach, a method previously developed to extract incidence-based mortality data collected by the cancer registry.(5) Since the dataset only contained the year of diagnosis and rounded survival time, we adopted an approach commonly used in calculating incidence rates (1) to determine mortality cases which were then used to calculate prevalence at a specific time point (i.e., end of 2009). We assumed that all new patients were diagnosed at the same time within a specific year (e.g., mid-2009). Consequently, patients who contributed 0.5 years of follow-up time and subsequently died were counted as mortality cases in that specific year (e.g., 2009), while patients who contributed a full year of follow-up time and then died were accounted for in the following year mortality (i.e., 2010).

# Supporting Table 2. Incidence rate per 10^7^ person-years by age group

|  |  | **CLL/SLL** | | | | | | | **WM** | | | | | | | | | | | | | | | | |  |  |  |  |
| --- | --- | --- | --- | --- | --- | --- | --- | --- | --- | --- | --- | --- | --- | --- | --- | --- | --- | --- | --- | --- | --- | --- | --- | --- | --- | --- | --- | --- | --- |
| **Year** | **Age group, y** | **Cases** | **Male IR** | **Cases** | **Female IR** | **Cases** | **Total IR** | **Cases** | | **Male IR** | **Cases** | | | | **Female IR** | | | | | **Cases** | | **Total IR** | | |  |  |  |  |  |
| 2009 | <50^#*^ | 29 | 96.78 | 20 | 67.65 | 49 | 82.31 | - | | - | - | | | | - | | | | | - | | - | | |  |  |  |  |  |
|  | 50-59^%*^ | 61 | 939.28 | 46 | 693.88 | 107 | 815.32 | - | | 14.05 | - | | | | 13.77 | | | | | 6 | | 13.91 | | |  |  |  |  |  |
|  | 60-69 | 134 | 2 738.67 | 48 | 975.51 | 182 | 1 854.61 | 13 | | 265.69 | 6 | | | | 121.94 | | | | | 19 | | 193.61 | | |  |  |  |  |  |
|  | 70-79 | 127 | 4 556.04 | 69 | 2 246.61 | 196 | 3 345.39 | 15 | | 538.11 | 6 | | | | 195.36 | | | | | 21 | | 358.43 | | |  |  |  |  |  |
|  | 80+ | 87 | 6 024.35 | 75 | 3 250.58 | 162 | 4 318.36 | 7 | | 484.72 | 11 | | | | 476.75 | | | | | 18 | | 479.82 | | |  |  |  |  |  |
| 2010 | <50^#*^ | 30 | 99.15 | 17 | 56.84 | 47 | 78.12 | - | | - | - | | | | - | | | | | - | | - | | |  |  |  |  |  |
|  | 50-59^%*^ | 80 | 1 210.25 | 43 | 635.91 | 123 | 919.82 | - | | 18.53 | 5 | | | | 22.69 | | | | | 9 | | 20.63 | | |  |  |  |  |  |
|  | 60-69 | 148 | 2 915.85 | 76 | 1 482.69 | 224 | 2 195.75 | 10 | | 197.02 | - | | | | - | | | | | 10 | | 98.02 | | |  |  |  |  |  |
|  | 70-79 | 140 | 4 880.21 | 65 | 2 075.37 | 205 | 3 416.27 | 10 | | 348.59 | 7 | | | | 223.5 | | | | | 17 | | 283.3 | | |  |  |  |  |  |
|  | 80+ | 84 | 5 579.84 | 100 | 4 213.56 | 184 | 4 743.85 | 11 | | 730.69 | 5 | | | | 210.68 | | | | | 16 | | 412.51 | | |  |  |  |  |  |
| 2011 | <50* | 23 | 75.52 | 12 | 39.77 | 35 | 57.73 | - | | - | - | | | | - | | | | | - | | - | | |  |  |  |  |  |
|  | 50-59% | 77 | 1 140.41 | 39 | 563.83 | 116 | 848.64 | - | | 13.72 | - | | | | 17.91 | | | | | 7 | | 15.84 | | |  |  |  |  |  |
|  | 60-69 | 150 | 2 856.66 | 59 | 1 107.55 | 209 | 1 975.81 | 9 | | 171.4 | 6 | | | | 112.63 | | | | | 15 | | 141.8 | | |  |  |  |  |  |
|  | 70-79 | 149 | 5 031.98 | 74 | 2 312.33 | 223 | 3 619.37 | 12 | | 405.26 | 5 | | | | 156.24 | | | | | 17 | | 275.92 | | |  |  |  |  |  |
|  | 80+ | 78 | 5 001.44 | 100 | 4 112.30 | 178 | 4 459.72 | 11 | | 705.33 | 7 | | | | 287.86 | | | | | 18 | | 450.98 | | |  |  |  |  |  |
| 2012 | <50^#*^ | 33 | 107.01 | 14 | 45.77 | 47 | 76.52 | - | | - | - | | | | - | | | | | - | | - | | |  |  |  |  |  |
|  | 50-59^%*^ | 67 | 972.35 | 37 | 522.13 | 104 | 744.09 | 6 | | 27 | 5 | | | | 22.01 | | | | | 11 | | 24.48 | | |  |  |  |  |  |
|  | 60-69 | 143 | 2 640.26 | 71 | 1 288.81 | 214 | 1 958.79 | 9 | | 166.17 | 6 | | | | 108.91 | | | | | 15 | | 137.3 | | |  |  |  |  |  |
|  | 70-79 | 139 | 4 518.47 | 67 | 2 023.43 | 206 | 3 225.06 | 10 | | 325.07 | 6 | | | | 181.2 | | | | | 16 | | 250.49 | | |  |  |  |  |  |
|  | 80+ | 99 | 6 147.81 | 79 | 3 192.45 | 178 | 4 357.49 | 10 | | 620.99 | 13 | | | | 525.34 | | | | | 23 | | 563.05 | | |  |  |  |  |  |
| 2013 | <50^#*^ | 30 | 96.08 | 11 | 35.49 | 41 | 65.9 | - | | - | - | | | | - | | | | | - | | - | | |  |  |  |  |  |
|  | 50-59^%*^ | 78 | 1 112.75 | 41 | 566.63 | 119 | 835.36 | 7 | | 31.02 | - | | | | 4.33 | | | | | 8 | | 17.52 | | |  |  |  |  |  |
|  | 60-69 | 159 | 2 840.85 | 76 | 1 329.68 | 235 | 2 077.34 | 14 | | 250.14 | - | | | | 6.83 | | | | | 17 | | 150.28 | | |  |  |  |  |  |
|  | 70-79 | 125 | 3 923.30 | 69 | 2 017.34 | 194 | 2 936.53 | 5 | | 156.93 | 8 | | | | 233.89 | | | | | 13 | | 196.78 | | |  |  |  |  |  |
|  | 80+ | 102 | 6 137.63 | 70 | 2 781.18 | 172 | 4 116.01 | 12 | | 722.07 | - | | | | 158.92 | | | | | 16 | | 382.89 | | |  |  |  |  |  |
| 2014 | <50* | 36 | 114.11 | 13 | 41.42 | 49 | 77.86 | - | | - | - | | | | - | | | | | - | | - | | |  |  |  |  |  |
|  | 50-59%* | 76 | 1 067.14 | 40 | 542.63 | 116 | 800.37 | 5 | | 21.89 | 5 | | | | 21.34 | | | | | 10 | | 21.61 | | |  |  |  |  |  |
|  | 60-69 | 151 | 2 634.63 | 87 | 1 477.46 | 238 | 2 048.22 | 14 | | 244.27 | 6 | | | | 101.89 | | | | | 20 | | 172.12 | | |  |  |  |  |  |
|  | 70-79 | 156 | 4 688.81 | 92 | 2 579.07 | 248 | 3 597.20 | 13 | | 390.73 | 8 | | | | 224.27 | | | | | 21 | | 304.6 | | |  |  |  |  |  |
|  | 80+ | 97 | 5 649.65 | 63 | 2 458.88 | 160 | 3 739.14 | 10 | | 582.44 | 6 | | | | 234.18 | | | | | 16 | | 373.91 | | |  |  |  |  |  |
| 2015 | <50^#*^ | 33 | 103.43 | 19 | 59.72 | 52 | 81.6 | - | | - | - | | | | - | | | | | - | | - | | |  |  |  |  |  |
|  | 50-59^%*^ | 82 | 1 139.77 | 41 | 548.64 | 123 | 838.59 | - | | 17.31 | 6 | | | | 25.22 | | | | | 10 | | 21.32 | | |  |  |  |  |  |
|  | 60-69 | 196 | 3 343.42 | 81 | 1 336.68 | 277 | 2 323.42 | 7 | | 119.41 | 8 | | | | 132.02 | | | | | 15 | | 125.82 | | |  |  |  |  |  |
|  | 70-79 | 174 | 4 996.12 | 110 | 2 954.88 | 284 | 3 941.52 | 17 | | 488.13 | 7 | | | | 188.04 | | | | | 24 | | 333.09 | | |  |  |  |  |  |
|  | 80+ | 129 | 7 299.64 | 96 | 3 688.17 | 225 | 5 148.59 | 11 | | 622.45 | 6 | | | | 230.51 | | | | | 17 | | 389 | | |  |  |  |  |  |
| 2016 | <50^#*^ | 32 | 98.77 | 20 | 61.75 | 52 | 80.26 | - | | - | - | | | | - | | | | | - | | - | | |  |  |  |  |  |
|  | 50-59^%*^ | 90 | 1 241.36 | 57 | 753.69 | 147 | 992.38 | - | | 17.09 | 8 | | | | 33.07 | | | | | 12 | | 25.21 | | |  |  |  |  |  |
|  | 60-69 | 196 | 3 270.04 | 118 | 1 889.95 | 314 | 2 565.91 | 11 | | 183.52 | 6 | | | | 96.1 | | | | | 17 | | 138.92 | | |  |  |  |  |  |
|  | 70-79 | 217 | 5 949.28 | 127 | 3 266.57 | 344 | 4 565.14 | 14 | | 383.82 | 8 | | | | 205.77 | | | | | 22 | | 291.96 | | |  |  |  |  |  |
|  | 80+ | 148 | 8 067.64 | 114 | 4 293.27 | 262 | 5 835.44 | 13 | | 708.64 | 13 | | | | 489.58 | | | | | 26 | | 579.09 | | |  |  |  |  |  |
| 2017 | <50^#*^ | 33 | 99.75 | 21 | 63.6 | 54 | 81.69 | - | | - | - | | | | - | | | | | - | | - | | |  |  |  |  |  |
|  | 50-59^%*^ | 130 | 1 776.77 | 54 | 706.09 | 184 | 1 229.59 | 7 | | 29.4 | - | | | | 12.19 | | | | | 10 | | 20.65 | | |  |  |  |  |  |
|  | 60-69 | 209 | 3 458.64 | 127 | 2 001.39 | 336 | 2 712.21 | 10 | | 165.49 | 8 | | | | 126.07 | | | | | 18 | | 145.3 | | |  |  |  |  |  |
|  | 70-79 | 234 | 6 022.67 | 155 | 3 757.24 | 389 | 4 856.01 | 19 | | 489.02 | 10 | | | | 242.4 | | | | | 29 | | 362.02 | | |  |  |  |  |  |
|  | 80+ | 164 | 8 655.40 | 126 | 4 656.82 | 290 | 6 303.69 | 25 | | 1 319.42 | 8 | | | | 295.67 | | | | | 33 | | 717.32 | | |  |  |  |  |  |
| 2018 | <50^#*^ | 33 | 97.84 | 17 | 50.53 | 50 | 74.22 | - | | - | - | | | | - | | | | | - | | - | | |  |  |  |  |  |
|  | 50-59^%*^ | 92 | 1 244.70 | 47 | 608.62 | 139 | 919.7 | 5 | | 20.66 | 6 | | | | 23.98 | | | | | 11 | | 22.35 | | |  |  |  |  |  |
|  | 60-69 | 206 | 3 362.85 | 96 | 1 481.42 | 302 | 2 395.68 | 23 | | 375.46 | 8 | | | | 123.45 | | | | | 31 | | 245.91 | | |  |  |  |  |  |
|  | 70-79 | 226 | 5 528.67 | 130 | 2 995.07 | 356 | 4 223.89 | 31 | | 758.36 | 18 | | | | 414.7 | | | | | 49 | | 581.38 | | |  |  |  |  |  |
|  | 80+ | 146 | 7 444.88 | 115 | 4 162.93 | 261 | 5 525.49 | 12 | | 611.91 | 12 | | | | 434.39 | | | | | 24 | | 508.09 | | |  |  |  |  |  |
|  | | | | | | | | | | | | | | | |  | | | | |  | |  | | | |  | |  |
| -*: cases are not reported due to small count (<5) | | | | | | | | | | | |  |  |  | |  |  |  |  | |  | |  | | | |  | |  |
| #: cumulative cases for the under 50 years age groups are shown for CLL/SLL and WM due to small counts (i.e., equal to one or under 5) presented in the lower age groups | | | | | | | | | | | | | | | | | | | | | | | | | | | | | |
| %: cumulative cases for WM in 30-49 group and 50-59 age groups due to no small counts (i.e., equal to one or under 5) presented in the lower age groups | | | | | | | | | | | | | | | | | | | | | | | |  | | | |  | |

CLL/SLL, chronic lymphocytic leukemia/small lymphocytic lymphoma; IR, incidence rate; WM, Waldenström macroglobulinemia.

# Supporting Table 3. Table of coefficient results for incidence rate examining the trend over 10 years observed data

|  | **CLL/SLL** | | | | | | **WM** | | | | | |
| --- | --- | --- | --- | --- | --- | --- | --- | --- | --- | --- | --- | --- |
|  | **Male** | | **Female** | | **Total** | | **Male** | | **Female** | | **Total** | |
|  | **Coefficients (SE)/AAPC** | **P-value** | **Coefficients (SE)/AAPC** | **P-value** | **Coefficients (SE)/AAPC** | **P-value** | **Coefficients (SE)/AAPC** | **P-value** | **Coefficients (SE)/AAPC** | **P-value** | **Coefficients (SE)/AAPC** | **P-value** |
|  | **(95% CI)** |  | **(95% CI)** |  | **(95% CI)** |  | **(95% CI)** |  | **(95% CI)** |  | **(95% CI)** |  |
| **Incidence- crude, least square linear regression** | | | | | | | | | | | | |
| **Constant (β_0_)** | 744.71 | <0.001 | 428.24 | <0.001 | 585.89 | <0.001 | 53.27 | <0.001 | 33.92 | 0.002 | 43.57 | <0.001 |
|  | -54.82 |  | -53.23 |  | -53.58 |  | -8.9 |  | -7.37 |  | -6.71 |  |
| **Year (β_1_)** | 44.83 | 0.001 | 26.91 | 0.014 | 35.67 | 0.003 | 4.17 | 0.02 | 2.32 | 0.087 | 3.23 | 0.018 |
|  | -8.84 |  | -8.58 |  | -8.64 |  | -1.43 |  | -1.19 |  | -1.08 |  |
| **Number included in analysis** | 10 | | 10 | | 10 | | 10 | | 10 | | 10 | |
| **R^2^** | 0.763 | | 0.552 | | 0.681 | | 0.514 | | 0.322 | | 0.527 | |
| **RSD** | 80.249 | | 77.927 | | 78.44 | | 13.031 | | 10.793 | | 9.828 | |
| **Incidence- ASD, least square linear regression** | | | | | | | | | | | | |
| **Constant (β_0_)** | 685.22 | <0.001 | 396.02 | <0.001 | 542.42 | <0.001 | 42.4 | 0.001 | 26.68 | 0.008 | 34.79 | 0.001 |
|  | 60.11 |  | 61.9 |  | 60.66 |  | 8.08 |  | 7.35 |  | 5.89 |  |
| **Year (β_1_)** | 30.29 | 0.013 | 22.75 | 0.047 | 26.98 | 0.023 | 3.59 | 0.023 | 2.66 | 0.05 | 3.20 | 0.009 |
|  | 9.2 |  | 9.48 |  | 9.29 |  | 1.24 |  | 1.12 |  | 0.9 |  |
| **Number included in analysis** | 10 | | 10 | | 10 | | 10 | | 10 | | 10 | |
| **R^2^** | 0.607 | | 0.452 | | 0.547 | | 0.545 | | 0.444 | | 0.527 | |
| **RSD** | 71.278 | | 73.4 | | 71.938 | | 9.587 | | 8.71 | | 6.983 | |
| **Incidence- ASD- joinpoint regression** | | | | | | | | | | | | |
| **APC, 0 joinpoint (1 segment)** | 3.56 | 0.006 | 4.39 | 0.049 | 3.91 | 0.034 | 5.23 | 0.011 | 4.35 | 0.115 | 4.99 | 0.011 |
|  | (1.18, 6.99) |  | (0.03, 11.37) |  | (0.37, 9.23) |  | (1.52, 9.08) |  | (–1.54, 13.17) |  | (1.45, 8.66) |  |
| **APC, 1 joinpoint (2 segment S1 and S2)** | S1: –0.13(–11.36, 12.53) | 0.979 | S1: –1.44(–19.86, 21.21) | 0.864 | S1: –0.57(–14.45,15.55) | 0.926 | S1: –2.03(–10.15, 1.47) | 0.232 | S1: –0.43(–13.70, 11.88) | 0.941 | S1: –1.49(–15.22, 3.25) | 0.366 |
|  | S2:5.81 (– 0.76, 12.80) | 0.073 | S2: –8.14 (–3.03, 20.60) | 0.124 | S2:6.69 (–1.51, 15.58) | 0.092 | S2: 18.97 (10.79, 33.57) | <0.001 | S2: 13.35 (–10.45, 43.47) | 0.124 | S2:17.24 (8.30, 35.21) | <0.001 |
| **AAPC, 1 joinpoint (2 segment S1 and S2)** | 3.13 | 0.215 | 3.77 | 0.388 | 3.4 | 0.284 | 4.52 | <0.001 | 3.96 | 0.114 | 4.39 | <0.001 |
|  | (–1.78, 8.27) |  | (–4.59, 12.86) |  | (–4.59, 12.86) |  | (2.85, 6.63) |  | (–5.38, 14.23) |  | (2.11, 7.41) |  |
| **BIC (weighted) of the joinpoint regression*** | | | | | | | | | | | | |
| **0 joinpoint** | 15.0356117 |  | 14.5365122 |  | 15.5103568 |  | 8.702851 |  | 7.8055646 |  | 8.7416457 |  |
| **1 joinpoint** | 15.2379036 |  | 14.7810463 |  | 15.7307555 |  | 7.5285377 |  | 7.9805178 |  | 7.8159995 |  |

*Final joinpoint regression model was selected based on the BIC, with lower BIC indicating a better fit.

RSD, residual standard deviation; AAPC: average annual percentage change; SE: Standard error; CI: Confidence Interval S1: Segment 1 in the joinpoint regression; S2: Segment 1 in the joinpoint regression.

# Supporting Table 4. Thirty-year incidence prediction for CLL/SLL and WM

|  | **Using linear regression method** | | | | | | **using AAPC, derived from joinpoint regression^#^** | | | | | |
| --- | --- | --- | --- | --- | --- | --- | --- | --- | --- | --- | --- | --- |
|  | **CLL/SLL, cases per 10^7^ person-years** | | | **WM, cases per 10^7^ person-years** | | | **CLL/SLL, cases per 10^7^ person-years*** | | | **WM, cases per 10^7^ person-years*** | | |
| **Prediction year** | **Total** | **Male** | **Female** | **Total** | **Male** | **Female** | **Total** | **LCI** | **UCI** | **Total** | **LCI** | **UCI** |
| 1 | 839.23 | 1018.37 | 646.29 | 70.01 | 81.86 | 55.93 | 723.16 | 698.51 | 760.22 | 62.37 | 61.01 | 64.17 |
| 2 | 866.21 | 1048.66 | 669.04 | 73.21 | 85.45 | 58.59 | 751.42 | 701.07 | 830.41 | 65.11 | 62.29 | 68.93 |
| 3 | 893.20 | 1078.94 | 691.79 | 76.41 | 89.04 | 61.25 | 780.79 | 703.63 | 907.09 | 67.98 | 63.61 | 74.03 |
| 4 | 920.18 | 1109.23 | 714.55 | 79.61 | 92.62 | 63.91 | 811.30 | 706.21 | 990.84 | 70.96 | 64.95 | 79.52 |
| 5 | 947.16 | 1139.52 | 737.30 | 82.81 | 96.21 | 66.57 | 843.00 | 708.80 | 1082.33 | 74.08 | 66.32 | 85.41 |
| 6 | 974.14 | 1169.80 | 760.05 | 86.02 | 99.80 | 69.23 | 875.95 | 711.39 | 1182.27 | 77.34 | 67.72 | 91.74 |
| 7 | 1001.13 | 1200.09 | 782.80 | 89.22 | 103.39 | 71.89 | 910.18 | 714.00 | 1291.43 | 80.73 | 69.14 | 98.53 |
| 8 | 1028.11 | 1230.37 | 805.55 | 92.42 | 106.97 | 74.55 | 945.75 | 716.61 | 1410.67 | 84.28 | 70.60 | 105.83 |
| 9 | 1055.09 | 1260.66 | 828.31 | 95.62 | 110.56 | 77.21 | 982.71 | 719.24 | 1540.92 | 87.99 | 72.09 | 113.67 |
| 10 | 1082.07 | 1290.95 | 851.06 | 98.82 | 114.15 | 79.87 | 1021.12 | 721.87 | 1683.20 | 91.85 | 73.61 | 122.09 |
| 11 | 1109.06 | 1321.23 | 873.81 | 102.02 | 117.73 | 82.53 | 1061.02 | 724.51 | 1838.61 | 95.89 | 75.16 | 131.13 |
| 12 | 1136.04 | 1351.52 | 896.56 | 105.22 | 121.32 | 85.18 | 1102.49 | 727.17 | 2008.38 | 100.10 | 76.75 | 140.85 |
| 13 | 1163.02 | 1381.81 | 919.31 | 108.43 | 124.91 | 87.84 | 1145.57 | 729.83 | 2193.82 | 104.50 | 78.36 | 151.28 |
| 14 | 1190.00 | 1412.09 | 942.07 | 111.63 | 128.50 | 90.50 | 1190.34 | 732.50 | 2396.38 | 109.09 | 80.02 | 162.49 |
| 15 | 1216.99 | 1442.38 | 964.82 | 114.83 | 132.08 | 93.16 | 1236.86 | 735.19 | 2617.65 | 113.89 | 81.70 | 174.52 |
| 16 | 1243.97 | 1472.66 | 987.57 | 118.03 | 135.67 | 95.82 | 1285.19 | 737.88 | 2859.34 | 118.89 | 83.42 | 187.45 |
| 17 | 1270.95 | 1502.95 | 1010.32 | 121.23 | 139.26 | 98.48 | 1335.42 | 740.58 | 3123.35 | 124.11 | 85.18 | 201.34 |
| 18 | 1297.94 | 1533.24 | 1033.07 | 124.43 | 142.85 | 101.14 | 1387.61 | 743.29 | 3411.74 | 129.57 | 86.98 | 216.25 |
| 19 | 1324.92 | 1563.52 | 1055.82 | 127.63 | 146.43 | 103.80 | 1441.84 | 746.01 | 3726.76 | 135.26 | 88.81 | 232.27 |
| 20 | 1351.90 | 1593.81 | 1078.58 | 130.83 | 150.02 | 106.46 | 1498.18 | 748.75 | 4070.86 | 141.21 | 90.69 | 249.48 |
| 21 | 1378.88 | 1624.09 | 1101.33 | 134.04 | 153.61 | 109.12 | 1556.73 | 751.49 | 4446.73 | 147.41 | 92.60 | 267.96 |
| 22 | 1405.87 | 1654.38 | 1124.08 | 137.24 | 157.19 | 111.78 | 1617.57 | 754.24 | 4857.31 | 153.89 | 94.55 | 287.81 |
| 23 | 1432.85 | 1684.67 | 1146.83 | 140.44 | 160.78 | 114.44 | 1680.78 | 757.00 | 5305.80 | 160.65 | 96.54 | 309.13 |
| 24 | 1459.83 | 1714.95 | 1169.58 | 143.64 | 164.37 | 117.10 | 1746.47 | 759.77 | 5795.70 | 167.71 | 98.58 | 332.03 |
| 25 | 1486.81 | 1745.24 | 1192.34 | 146.84 | 167.96 | 119.76 | 1814.72 | 762.56 | 6330.84 | 175.08 | 100.66 | 356.62 |
| 26 | 1513.80 | 1775.52 | 1215.09 | 150.04 | 171.54 | 122.41 | 1885.64 | 765.35 | 6915.39 | 182.77 | 102.78 | 383.04 |
| 27 | 1540.78 | 1805.81 | 1237.84 | 153.24 | 175.13 | 125.07 | 1959.33 | 768.15 | 7553.90 | 190.80 | 104.95 | 411.42 |
| 28 | 1567.76 | 1836.10 | 1260.59 | 156.45 | 178.72 | 127.73 | 2035.90 | 770.96 | 8251.38 | 199.19 | 107.16 | 441.89 |
| 29 | 1594.74 | 1866.38 | 1283.34 | 159.65 | 182.31 | 130.39 | 2115.46 | 773.79 | 9013.25 | 207.94 | 109.42 | 474.63 |
| 30 | 1621.73 | 1896.67 | 1306.09 | 162.85 | 185.89 | 133.05 | 2198.14 | 776.62 | 9845.47 | 217.08 | 111.72 | 509.79 |

CLL/SLL, chronic lymphocytic leukemia/small lymphocytic lymphoma; WM, Waldenström macroglobulinemia.

*: using the lower bounce of the co-efficient

#: projected incidence was done using formulae: {Id*(AAPC/100+1)n for AAPC>0 and {Id*(|AAPC|/100+1)-n for AAPC<)} with Id is the average incidence rate at baseline (i.e, 10 years data, AAPC is the average annual percentage change and n is number of years elapsed since the end of the observed period (i.e., end of 2018)

Reference for the formula: https://pmc.ncbi.nlm.nih.gov/articles/PMC9614249/

# Supporting Table 5. Prevalence proportion per 10^7^ persons-years by sex and age group for CLL/SLL and WM between 2009-2018

|  | **2009** | **2010** | **2011** | **2012** | **2013** | **2014** | **2015** | **2016** | **2017** | **2018** |
| --- | --- | --- | --- | --- | --- | --- | --- | --- | --- | --- |
| CLL | | | | | | | | | | |
| Male age group, y | | | | | | | | | | |
| < 40-49* | 627.5 | 746.83 | 664.19 | 660.25 | 706.05 | 705.41 | 733.08 | 767.05 | 1 040.81 | 778.97 |
| 50-59 | 1 712.44 | 1 951.07 | 1 891.22 | 1 706.39 | 1 879.97 | 1 766.10 | 2 129.17 | 2 159.48 | 2 537.52 | 2 204.62 |
| 60-69 | 3 398.26 | 3 625.18 | 3 641.04 | 3 320.62 | 3 233.52 | 3 389.11 | 3 959.35 | 4 283.65 | 4 462.06 | 4 229.68 |
| 70-79 | 4 773.55 | 4 869.52 | 4 800.24 | 4 800.93 | 4 393.59 | 4 639.18 | 5 466.76 | 6 384.54 | 6 332.08 | 5 786.21 |
| 80-89 | 6 023.85 | 5 579.43 | 5 001.12 | 6 147.31 | 6 137.11 | 5 649.03 | 7 298.98 | 8 066.98 | 8 653.94 | 7 444.04 |
| ASD | 991.13 | 1053.23 | 1011.29 | 997.13 | 991.97 | 1001.07 | 1170.13 | 1270.72 | 1378.98 | 1 225.00 |
| Female age group, y | | | | | | | | | | |
| 40-49* | 458.21 | 409.7 | 327.09 | 313.85 | 341.79 | 330.76 | 378.48 | 469.91 | 440.48 | 387.01 |
| 50-59 | 813.86 | 1 001.03 | 800.39 | 857.46 | 903.38 | 957.77 | 901.51 | 1 267.53 | 1 293.48 | 1 006.85 |
| 60-69 | 1 464.00 | 1 707.48 | 1 559.70 | 1 564.60 | 1 587.13 | 1 893.05 | 1 952.48 | 2 418.22 | 2 693.17 | 2 088.58 |
| 70-79 | 2 472.77 | 2 579.35 | 2 663.37 | 2 281.45 | 2 105.35 | 2 365.68 | 3 051.11 | 3 285.86 | 3 689.02 | 3 167.70 |
| 80-89 | 3 250.43 | 4 213.15 | 4 111.89 | 3 192.27 | 2 781.02 | 2 458.79 | 3 687.98 | 4 292.85 | 4 656.32 | 4 162.63 |
| ASD | 581.79 | 657.94 | 609.03 | 556.07 | 533.24 | 568.97 | 670.86 | 804.11 | 866.27 | 719.10 |
| WM | | | | | | | | | | |
| Male age group, y |  |  |  |  |  |  |  |  |  |  |
| <50* | - | - | - | - | - | - | - | - | - | - |
| 50-59 | 140.51 | 111.25 | 83.31 | 113.76 | 166.58 | 132.26 | 84.25 | 105.71 | 119.77 | 207.15 |
| 60-69 | 364.56 | 251.75 | 255.73 | 223.73 | 216.33 | 298.06 | 256.82 | 259.3 | 292.1 | 528.71 |
| 70-79 | 496.26 | 411.51 | 508.78 | 405.41 | 350.66 | 416.34 | 514.29 | 474.28 | 674.73 | 710.88 |
| 80-89 | 484.71 | 730.68 | 705.33 | 620.99 | 722.07 | 582.43 | 622.45 | 708.64 | 1319.39 | 611.91 |
| ASD | 86.52 | 76.90 | 78.55 | 74.15 | 79.07 | 81.76 | 76.05 | 79.43 | 110.74 | 123.56 |
| Female age group, y |  |  |  |  |  |  |  |  |  |  |
| <50^#^ | - | - | - | - | - | - | - | - | - | - |
| 50-59 | 77.92 | - | 73.51 | 79.39 | - | 52.79 | 88.67 | 86.92 | 71.46 | 84.49 |
| 60-69 | 150.15 | 84.77 | 129 | 136.05 | 120.4 | 148.06 | 153.34 | 138.18 | 171.9 | 240.28 |
| 70-79 | 278.88 | 217.97 | 177.56 | 276.54 | 185.27 | 212.1 | 205.52 | 290.38 | 263.5 | 408.28 |
| 80-89 | 476.75 | 210.68 | 287.86 | 525.33 | 158.92 | 234.18 | 230.51 | 489.58 | 295.67 | 434.39 |
| ASD | 60.45 | 38.85 | 46.25 | 64.06 | 32.00 | 48.96 | 51.96 | 67.99 | 53.83 | 78.95 |

^†^ Including 10-19, 20-29, and 30-39 age group.
^#^Including 10-19, 20-29, 30-39 and 40-49 age group.

- is not reported due to the rate being less than five per 10^7^ person/person-years.

# Supporting Table 6. Linear regression by age group for prevalence proportion of CLL/SLL and WM (per 10^7^ person-years) examining the trend over 10 years observed data

|  | **CLL/SLL** | | | | | | **WM** | | | | | |
| --- | --- | --- | --- | --- | --- | --- | --- | --- | --- | --- | --- | --- |
|  | **Male** | | | **Female** | | | **Male** | | | **Female** | | |
|  | Coefficient* | SE | p | Coefficient* | SE | p | Coefficient* | SE | p | Coefficient | SE | p |
| Year (β1) | 88.02 | 17.85 | <0.001 | 44.27 | 12.72 | 0 | 10.29 | 4.15 | 0.016 | 4.03 | 2.46 | 0.106 |
| 10 –19 | -6 597.01 | 205.06 | <0.001 | -3 678.74 | 146.14 | <0.001 |  |  |  |  |  |  |
| 20-29 | -6 571.69 | 205.06 | <0.001 | -3 668.13 | 146.14 | <0.001 | -710.29 | 44.56 | <0.001 | -334.39 | 26.41 | <0.001 |
| 30-39 | -6 404.73 | 205.06 | <0.001 | -3 580.64 | 146.14 | <0.001 | -705.72 | 44.56 | <0.001 | -324.47 | 26.41 | <0.001 |
| 40-49 | -5 857.17 | 205.06 | <0.001 | -3 295.01 | 146.14 | <0.001 | -679.03 | 44.56 | <0.001 | -304.27 | 26.41 | <0.001 |
| 50-59 | -4 606.38 | 205.06 | <0.001 | -2 700.41 | 146.14 | <0.001 | -584.41 | 44.56 | <0.001 | -268.10 | 26.41 | <0.001 |
| 60-69 | -2 845.93 | 205.06 | <0.001 | -1 787.89 | 146.14 | <0.001 | -416.15 | 44.56 | <0.001 | -187.17 | 26.41 | <0.001 |
| 70-79 | -1 375.52 | 205.06 | <0.001 | -914.57 | 146.14 | <0.001 | -214.55 | 44.56 | <0.001 | -82.79 | 26.41 | 0.003 |
| Constant (β0) | 6 116.07 | 175.1 | <0.001 | 3 437.26 | 124.79 | <0.001 | 654.27 | 38.90 | <0.001 | 312.24 | 23.05 | <0.001 |
| R^2^ | 0.969 | | | 0.949 | | | 0.883 | | | 0.826 | | |
| Root mean square error | 458.52 | | | 326.78 | | | 99.648 | | | 59.054 | | |
| ASD | | | | | | |  | | | | | |
| Constant (β0) | 870.62 | 77.90 | <0.001 | 500.06 | 77.47 | <0.001 | 57.61 | 10.55 | 0.001 | 32.56 | 9.98 | 0.014 |
| Year (β1) | 41.92 | 11.93 | 0.01 | 27.50 | 11.86 | 0.053 | 4.85 | 1.62 | 0.02 | 3.52 | 1.53 | 0.055 |
| R^2^ | 0.638 | | | 0.434 | | | 0.562 | | | 0.431 | | |
| Root mean square error | 92.37 | | | 91.86 | | | 12.51 | | | 9.98 | | |

^†^ 80-89 is the reference group; ASD: Age standardised; SE: standard errors

# Supporting Figure 1A. Joinpoint regression result CLL/SLL

| **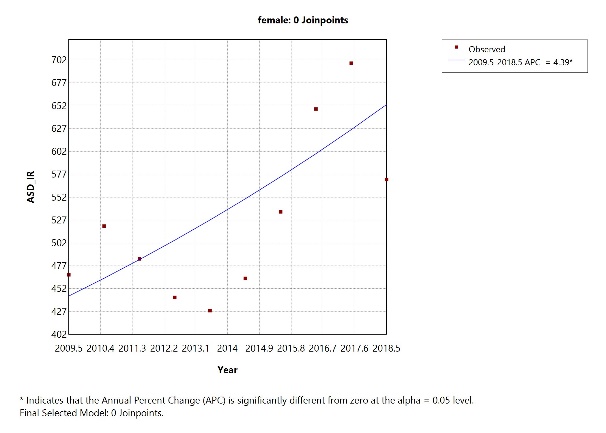** | **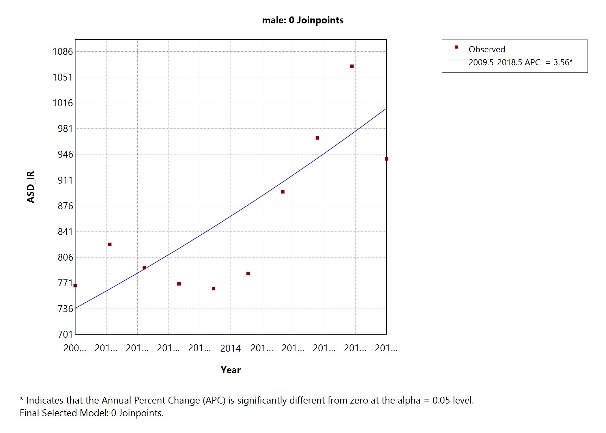** | **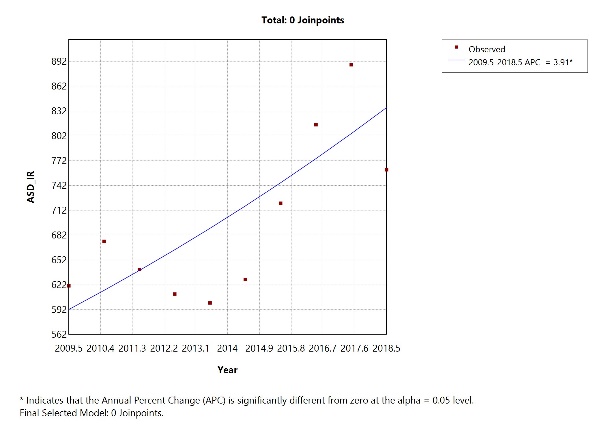** |
| --- | --- | --- |

Note: The final model for females, males and total persons is one segment (0 joinpoint)

##

## Supporting Figure 1B. Joinpoint regression result WM

| **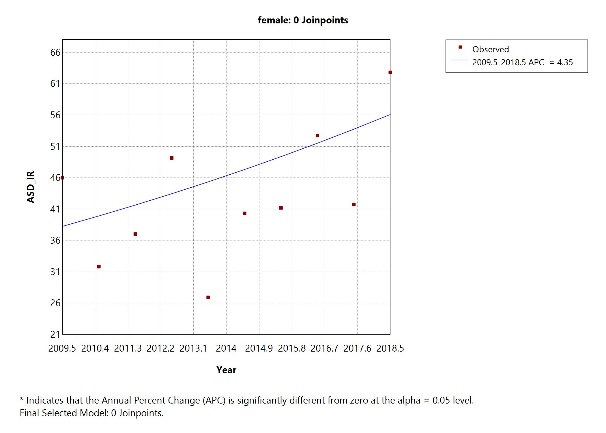** | **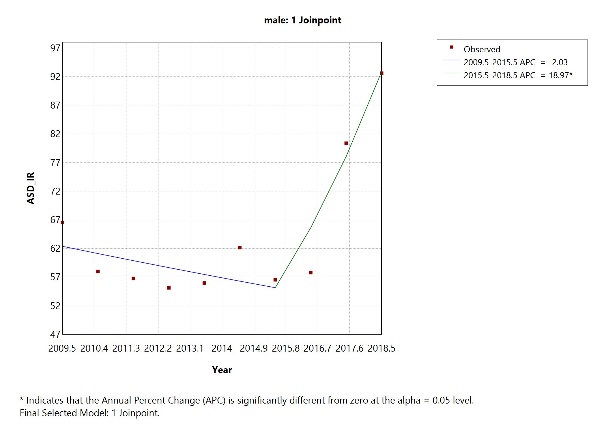** | **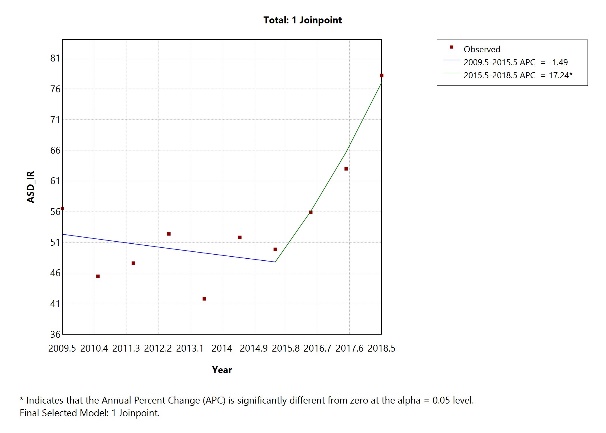** |
| --- | --- | --- |

Note: The final model for females is one segment (0 joinpoint) while the final model for males and total persons is two segments (1 joinpoint)

# Supporting Figure 2. Kaplan-Meier survival estimate by age group for CLL/SLL with 10 years of follow-up


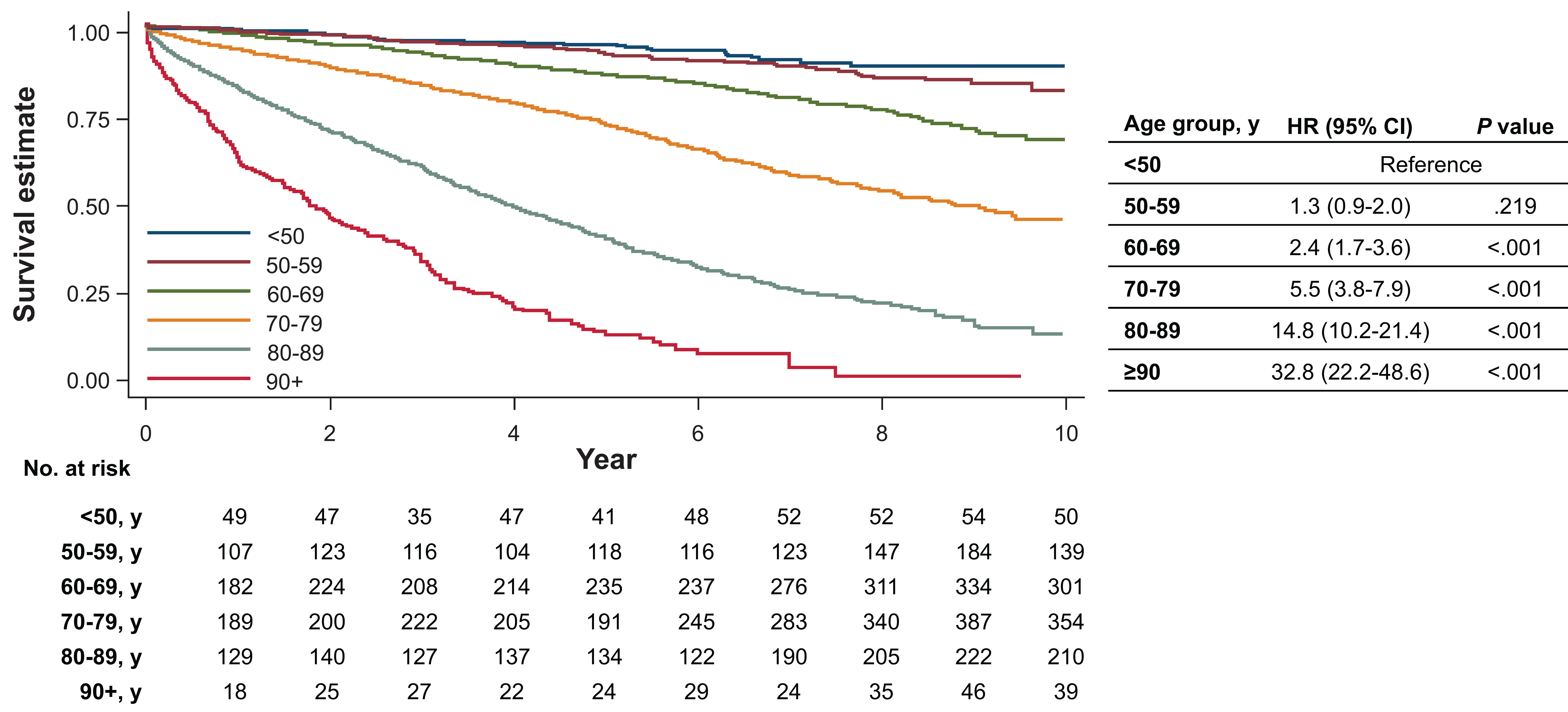


CLL/SLL, chronic lymphocytic leukemia/small lymphocytic lymphoma; HR, hazard ratio.

Curves were generated by Kaplan-Meier survival analyses and evaluated by the log-rank test (significance at p<0.05).

# Supporting Figure 3. Kaplan-Meier survival estimate by age group for WM with 10 years of follow-up


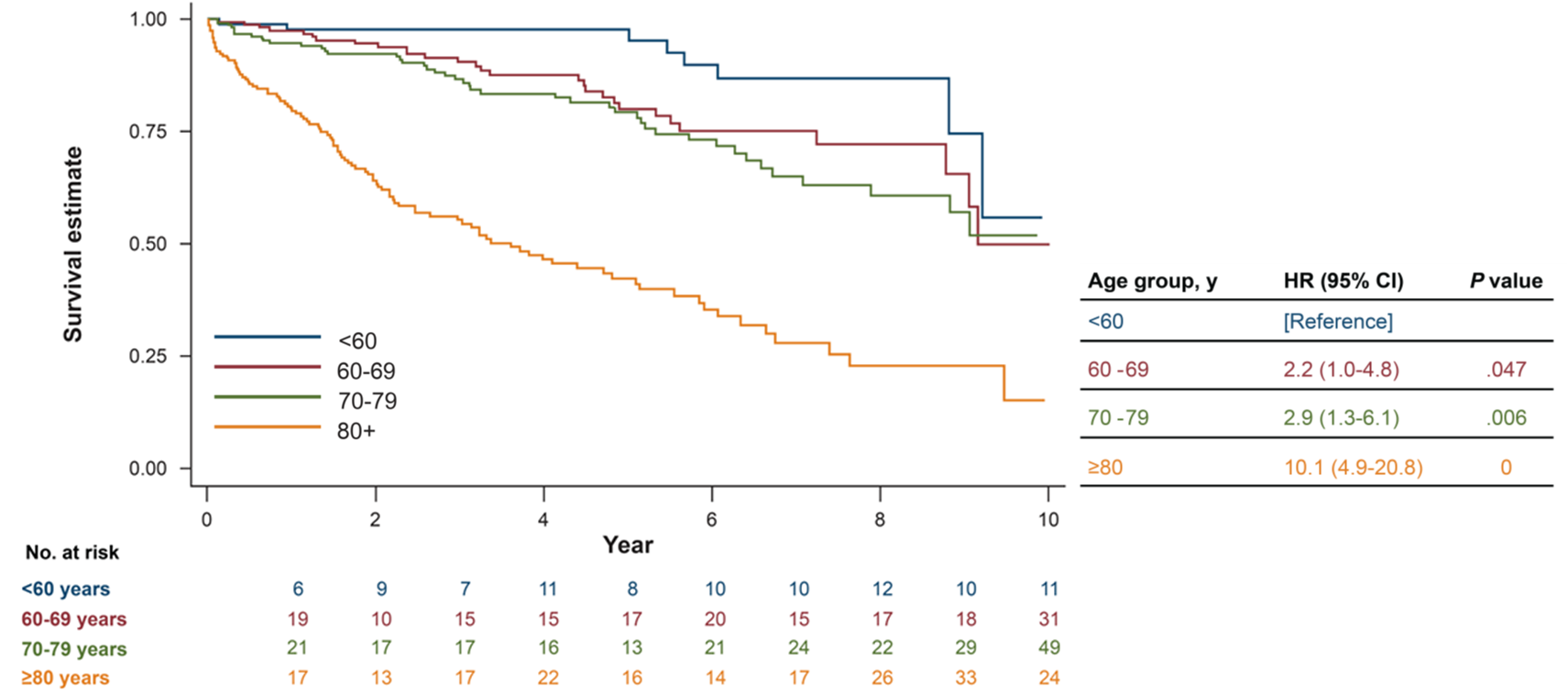


HR, hazard ratio; WM, Waldenström macroglobulinemia.

Curves were generated by Kaplan-Meier survival analyses and evaluated by the log-rank test (significance at p<0.05).

## Supporting Figure 4A. Schoenfeld and scaled Schoenfeld residual test for the proportionality assumption (CLL/SLL)

| **Proportional hazard: age group (all-cause mortality)** | **Proportional hazard: age group (cancer-specific mortality)** | **Proportional hazard: sex (all-cause mortality)** | **Proportional hazard: sex (cancer-specific mortality)** |
| --- | --- | --- | --- |
| **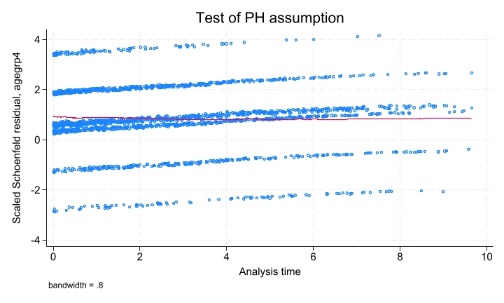** | **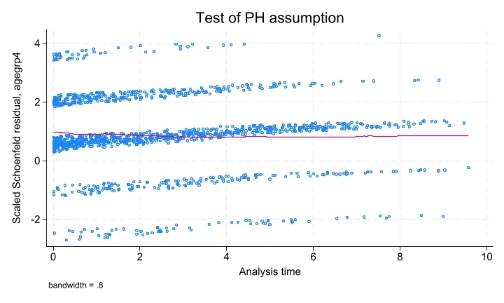** | **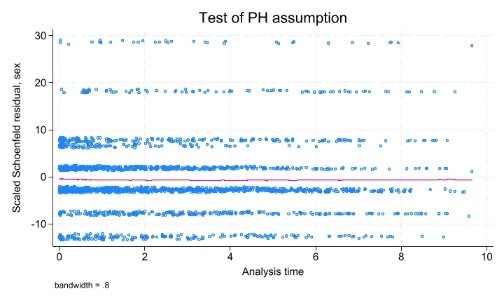** | **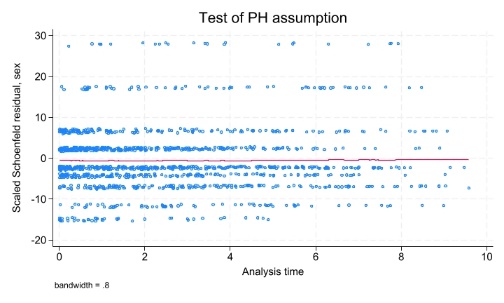** |
| **Stphplot: age group (all-cause mortality)** | **Stphplot: age group (cancer specific mortality)** | **Proportional hazard: year (all-cause mortality)** | **Proportional hazard: year(cancer-specific mortality)** |
| **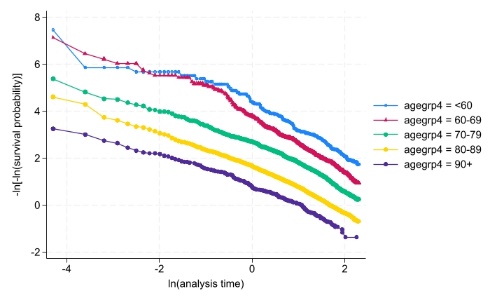** | **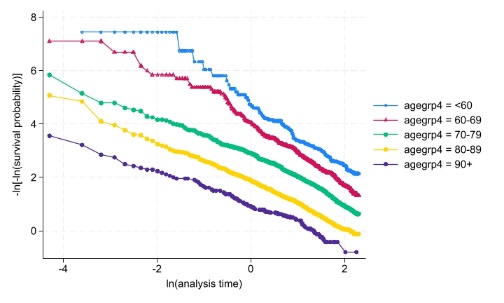** | **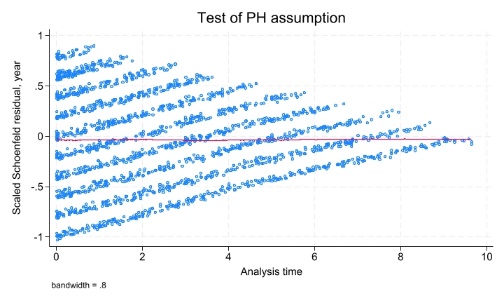** | **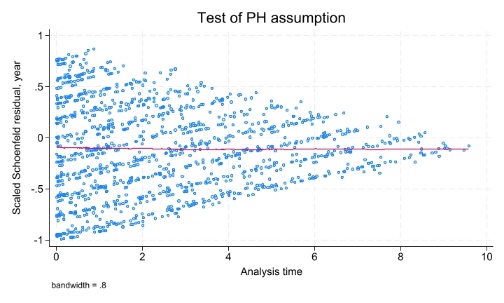** |
| **Stphplot: sex (all-cause mortality)** | **Stphplot: age group (cancer specific mortality)** |  |  |
| **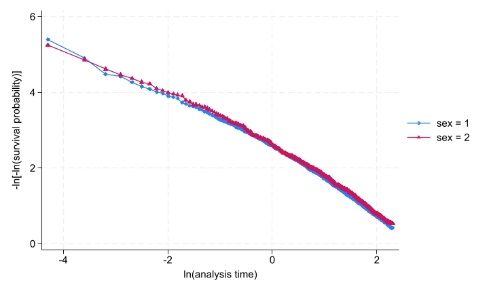** | **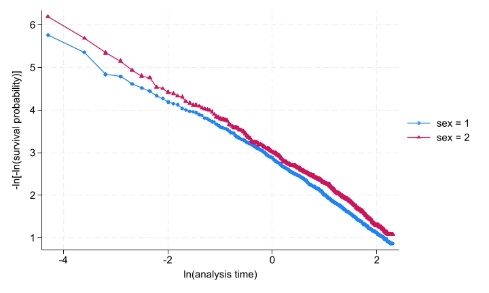** |  |  |

## Note: If a horizontal red line is presented in the test of the PH assumption graphs and the log-log plots show parallel lines, it suggests no proportionality violation. However, irregular patterns in the year of diagnosis indicated possible violation of the proportional hazard assumption or heteroskedasticity. A suitable modelling approach, along with a pre-estimation and post-estimation test, was employed to address this potential issue.

## Supporting Figure 4B. Schoenfeld and scaled Schoenfeld residual test for the proportionality assumption (WM)

| **Proportional hazard: age group (all-cause mortality)** | **Proportional hazard: age group (cancer-specific mortality)** | **Proportional hazard: sex (all-cause mortality)** | **Proportional hazard: sex (cancer-specific mortality)** |
| --- | --- | --- | --- |
| **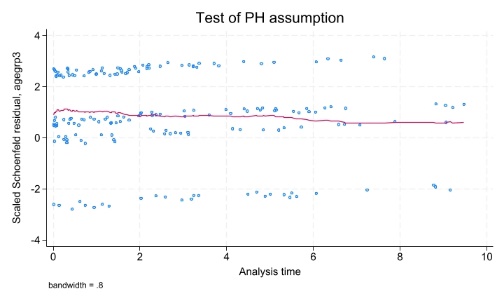** | **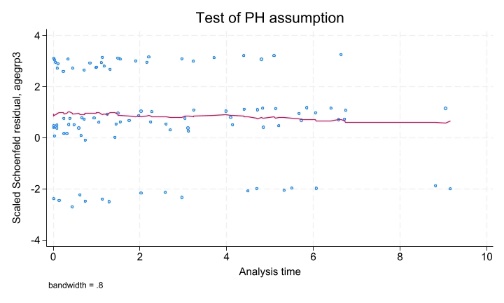** | **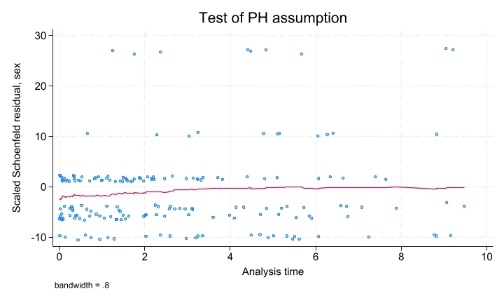** | **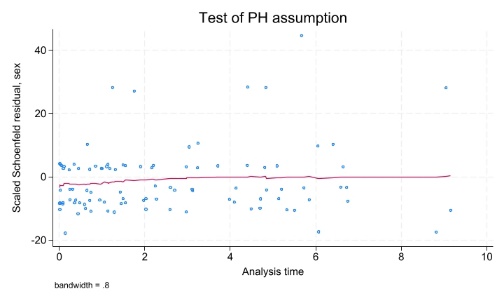** |
| **Stphplot: age group (all-cause mortality)** | **Stphplot: age group (cancer specific mortality)** | **Proportional hazard: year (all-cause mortality)** | **Proportional hazard: year(cancer-specific mortality)** |
| **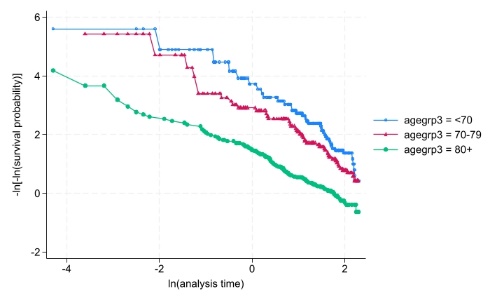** | **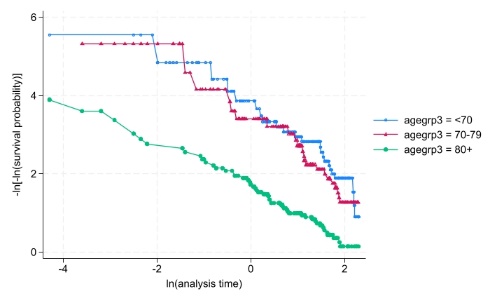** | **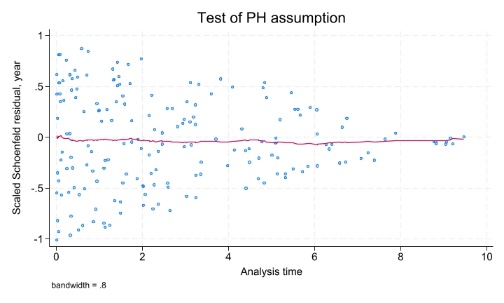** | **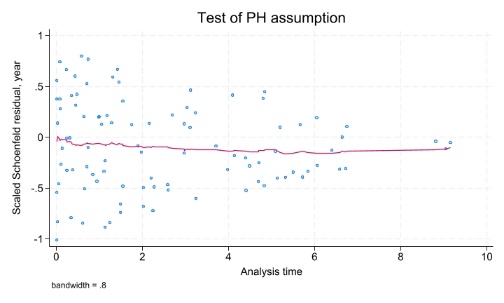** |
| **Stphplot: sex (all-cause mortality)** | **Stphplot: sex (cancer specific mortality)** |  |  |
| **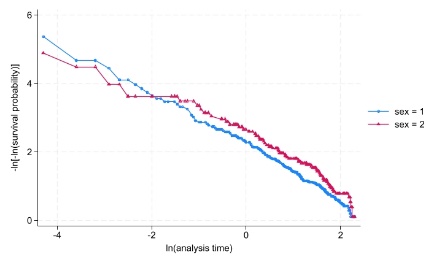** | **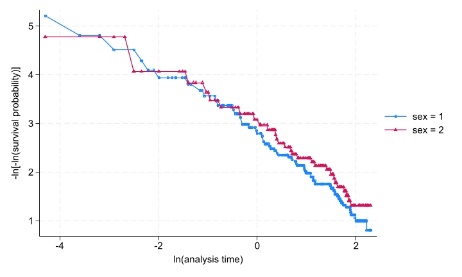** |  |  |

## Note: If a horizontal red line is presented in the test of the PH assumption graphs and the log-log plots show parallel lines, it suggests no proportionality violation. However, irregular patterns in the year of diagnosis indicated possible violation of the proportional hazard assumption or heteroskedasticity. A suitable modelling approach, along with a pre-estimation and post-estimation test, was employed to address this potential issue.

**References**

1. Webb P, Bain C, Page A. Essential epidemiology: an introduction for students and health professionals: Cambridge University Press, Cambridge; 2016.

2. Australian Institute for Health and Welfare. Principles on the use of direct age-standardisation in administrative data collections: for measuring the gap between Indigenous and non-Indigenous Australians 2011 [Available from: <https://www.aihw.gov.au/reports/indigenous-australians/principles-on-the-use-of-direct-age-standardisatio/summary>.

3. Barendregt JJ, Van Oortmarssen GJ, Vos T, Murray CJL. A generic model for the assessment of disease epidemiology: the computational basis of DisMod II. Popul Health Metr. 2003;1(4):1478-7954 (Print).

4. Australian Institute of Health and Welfare. Cancer data in Australia 2023 [Available from: <https://www.aihw.gov.au/reports/cancer/cancer-data-in-australia/contents/technical-notes/methods>.

5. Chu KC, Miller BA, Feuer EJ, Hankey BF. A method for partitioning cancer mortality trends by factors associated with diagnosis: an application to female breast cancer. J Clin Epidemiol. 1994;47(12):1451-61.
